# A new class of antibacterials, the imidazopyrazinones, reveal structural transitions involved in DNA gyrase poisoning and mechanisms of resistance

**DOI:** 10.1093/nar/gky181

**Published:** 2018-03-10

**Authors:** Thomas Germe, Judit Vörös, Frederic Jeannot, Thomas Taillier, Robert A Stavenger, Eric Bacqué, Anthony Maxwell, Benjamin D Bax

**Affiliations:** 1 Department of Biological Chemistry, John Innes Centre, Norwich Research Park, Norwich NR4 7UH, UK; 2 Sanofi R&D, TSU Infectious Diseases, 1541 Avenue Marcel Mérieux, 69280 Marcy L’Etoile, France; 3 Antibacterial Discovery Performance Unit, Infectious Diseases Therapy Area Unit, GlaxoSmithKline, 1250 Collegeville Road, Collegeville, PA 19426, USA; 4 Platform Technology and Science, GlaxoSmithKline, Medicines Research Centre, Gunnels Wood Road, Stevenage, Hertfordshire SG1 2NY, UK

## Abstract

Imidazopyrazinones (IPYs) are a new class of compounds that target bacterial topoisomerases as a basis for their antibacterial activity. We have characterized the mechanism of these compounds through structural/mechanistic studies showing they bind and stabilize a cleavage complex between DNA gyrase and DNA (‘poisoning’) in an analogous fashion to fluoroquinolones, but without the requirement for the water–metal–ion bridge. Biochemical experiments and structural studies of cleavage complexes of IPYs compared with an uncleaved gyrase–DNA complex, reveal conformational transitions coupled to DNA cleavage at the DNA gate. These involve movement at the GyrA interface and tilting of the TOPRIM domains toward the scissile phosphate coupled to capture of the catalytic metal ion. Our experiments show that these structural transitions are involved generally in poisoning of gyrase by therapeutic compounds and resemble those undergone by the enzyme during its adenosine triphosphate-coupled strand-passage cycle. In addition to resistance mutations affecting residues that directly interact with the compounds, we characterized a mutant (D82N) that inhibits formation of the cleavage complex by the unpoisoned enzyme. The D82N mutant appears to act by stabilizing the binary conformation of DNA gyrase with uncleaved DNA without direct interaction with the compounds. This provides general insight into the resistance mechanisms to antibiotics targeting bacterial type II topoisomerases.

## INTRODUCTION

Antibiotics are a cornerstone of modern medicine, underpinning modern clinical and ambulatory care. The ability to prevent and treat infections has led to a huge drop in mortality and morbidity, and indirectly allowed the development of sophisticated surgical procedures. Few discoveries can claim to have had such an all-encompassing and far-reaching effect on human well being. Unfortunately the levels of antimicrobial resistance (AMR) worldwide are increasing (1,2) while recent decades have seen a paucity of new antibacterial compounds reaching the clinic (3) or approved for use (4). On this, see also the material published by the review on AMR commission chaired by Jim O’Neil, https://amr-review.org/Publications.html. The current perceived low return on investment in antibacterials does not encourage significant investment by industry. Moreover, the discovery and optimization of new antibacterials is intrinsically challenging and clinical trials face challenges as well (5).

Altogether, these issues create a potential ‘perfect storm’ wherein bacterial infections are becoming untreatable with traditional antibiotics and only limited new options are becoming available (6). Among the numerous counter attacks to fight AMR, the New Drugs For Bad Bugs (ND4BB) program, funded by the European Union’s Innovative Medicine Initiative (IMI), includes multiple collaborative projects, involving both pharmaceutical industries and academic partners, to accelerate the discovery and development of new treatments for resistance to bacterial infections (7,8). Within ND4BB, the ENABLE consortium tackles optimization and pre-clinical development of new compounds targeting Gram-negative pathogens by pooling the expertise of multiple partners to progress early stage drug discovery projects to key milestones.

DNA topoisomerases are enzymes responsible for the control of DNA topology in all cells and are classified into two types, I and II, dependent on whether their reactions proceed via single- or double-stranded DNA breaks (9,10). The bacterial type II topoisomerases, topoisomerase IV and DNA gyrase, are made up of two subunits forming hetero-tetrameric complexes, which establish a transient double-stranded DNA break and facilitate the passage of another DNA segment through the break. DNA gyrase, which consists of two subunits, GyrA and GyrB, forming an A_2_B_2_ complex in the active enzyme, wraps a positive turn of DNA around itself before the strand-passage event, thereby introducing negative supercoils. Bacterial type II topoisomerases are one of only a few clinically-validated targets for antibiotics (11). Type II topoisomerases are essential for transcription and genome segregation in bacterial cells and are the target of a successful family of antibiotics: the fluoroquinolones (FQs) (11,12). The FQs intercalate at the sites of DNA cleavage (the DNA gate), one molecule at each of the two cleavage sites, separated by four bases. This intercalation stabilizes a cleaved DNA complex (dubbed the ‘cleavage complex’) thereby converting the topoisomerase from an essential enzyme into a DNA-damaging agent. FQs and similar agents are therefore called topoisomerase ‘poisons’. The huge clinical success of these compounds has been undermined by the emergence of resistance, notably by mutations within the target itself (13). FQs interact, via a water–metal–ion bridge (14–16), with two residues on the GyrA subunit of DNA gyrase that are often mutated in FQ-resistant strains of bacteria found in the clinic (14,17–18).

Recently, a variety of compounds targeting bacterial type II topoisomerases have been reported, including two classes that have completed phase II trials (19–21). Bacterial type II topoisomerases can be targeted in various ways through several binding pockets. However, poisoning remains by far the most clinically successful way to target type II topoisomerases.

As part of a collaboration between Sanofi and GSK within the ENABLE consortium, the imidazopyrazinones (IPYs), a series of bactericidal compounds initially discovered by Rhône-Poulenc in an SOS activation assay, have been the subject of a medicinal chemistry effort for their pre-clinical optimization (Jeannot *et al.* In revision in J Med Chem). To support this effort, structural and mechanistic studies were performed. The most potent examples of this IPY series were the result of a structural hybridization between the initial, Gram-positive-only hits and the quinazolinedione series of topoisomerase inhibitors (22). In quinazolinediones, the carboxylic acid group of FQs is replaced with an amine, and no water–metal–ion bridge is involved in compound binding to DNA gyrase (23). During the microbiological and enzymatic profiling of the IPYs, partial cross resistance with the FQs was detected leading to the termination of the optimization effort (Jeannot *et al.* In revision in J Med Chem). We have now solved the crystal structure of selected IPYs bound to DNA gyrase and DNA and show that these IPYs bind the FQ pocket in a fashion similar to the FQs, despite the lack of the highly characteristic 4-oxo and carboxylic acid moieties of FQs. IPYs establish contact with a FQ-resistance-associated residue, thus explaining the observed cross resistance. Through comparison with a compound-free ‘unpoisoned’ binary gyrase–DNA complex and biochemical experiments, we further show that conformational changes associated with IPY-induced poisoning are highly similar to those observed with FQs and fit into a general ‘intercalative-poisoning’ scheme. This provides general insights into this major targeting avenue for antibiotics and the mechanism of target-based resistance to topoisomerase inhibitors. We discuss the role of the metal ions in the poisoned complex, potential strategies for the development of new type II topoisomerase poisons and the basis for resistance.

## MATERIALS AND METHODS

### Compound synthesis

Synthetic details for the IPYs are described elsewhere (Jeannot *et al.* In revision in J Med Chem), except for compound **b1**, whose synthesis and characterization is described in the ‘Supplementary Data’ section.

### Supercoiling assays

Supercoiling reactions were carried out as described previously (24) with minor adjustments. A total of 500 ng of relaxed pBR322 plasmid was used as a substrate for each 30 μl reaction. The amount of DNA gyrase (A_2_B_2_) added was normalized by testing various dilutions of the stock (A_2_B_2_ containing 0.5 mg/ml of each subunit) without compound. The amount sufficient to supercoil 50% of the substrate was then used for the testing of compounds. See Supplementary Figure S1 for details. This ensured a limiting amount of enzyme for every mutant tested. The individual gyrase subunits were either prepared in the lab as described previously (25) or purchased from Inspiralis. The IC_50_ (compound concentration giving only 50% of the supercoiled substrate obtained with the uninhibited enzyme) was determined by plotting the quantified (using ImageJ) proportion of supercoiled DNA to the total of the lane against the compound concentration and fitting it to a four-parameter binding curve (y = min + ((max − min)/(1 + (x/IC50)∧HillSlope)) with Scipy (26). The measured value was the best fit for the IC_50_ parameter. Comparing our measurement with ciprofloxacin to published results showed overall consistency and validated our methodology (for instance compare our ciprofloxacin data with those reported in (27)).

### Cleavage assays

Reactions were carried out as described previously (24) with minor adjustments. A total of 500 ng of relaxed pBR322 plasmid was used as a substrate for each 30 μl reaction. Cleavage complexes were trapped by addition of 7.5 μl of 1% (w/v) sodium dodecyl sulphate (SDS). Dimethylsulfoxide (DMSO) was absent (Ca^2+^ re-sealing), or at a concentration of 0.33% (v/v) (ciprofloxacin or **t1** resealing) or 1.66% (v/v) (all other cleavage experiments). When analyzing cleavage, electrophoresis was performed in the presence 0.5 μg/ml ethidium bromide. In these conditions, intact DNA molecules migrate as a single band (topoisomers are not resolved). For the ethylenediaminetetraacetic acid (EDTA) re-sealing experiment, 1 μl of a 250 mM stock of EDTA pH 8.0 was added prior to SDS trapping as above (after the indicated time). The CC_50_ was determined exactly as the IC_50_ and is defined as the concentration of compound producing 50% of the maximum cleavage obtained in the same assay.

### Crystallization and data collection

Crystals of DNA–cleavage complexes of two of the IPYs, **t1** and **t3**, with *Staphylococcus aureus* DNA gyrase fusion truncate (GyrB27-A56(GKdel)) were obtained as described (28). A crystal of **t1** gave a 3.06 Å dataset in a P2_1_ cell with two complexes in the asymmetric unit, while a crystal of **t3** gave a 3.11 Å dataset in a P6_1_ cell with one complex in the asymmetric unit (Supplementary Table S1). We also obtained a 2.60 Å structure of a binary complex of DNA and with the same *S. aureus* DNA gyrase core construct in a P2_1_ cell with two complexes in the asymmetric unit. The sequences of the 20 bp DNA duplexes used to obtain the structures are shown in Supplementary Table S2. The DNA used in the binary complex (20-447T), has previously been used in solving two crystal structures of cleavage complexes with QPT-1 (28). The DNA used in the two IPY complexes, 20-448T-U, contained an A-U base-pair (Supplementary Table S2) where the DNA is bent by the insertion of Ile 175 (from *S. aureus* GyrA).

### Structure determination and refinement

The structures were determined from previous crystal structures of *S. aureus* DNA gyrase (19,21,29–30). Structures were refined with cycles of rebuilding in Coot (31) and refinement in Refmac (32), Phenix (33) and BUSTER (BUSTER version 2.11.5; Global Phasing Ltd: Cambridge, UK) to give models with good geometry (Supplementary Table S1). Refinement dictionaries for the two IPYs, **t1** and **t3**, were generated with AceDRG (34). In the 2.60 Å binary structure the DNA was intact, whereas in the two IPY structures the DNA had been cleaved and there was a covalent phosphotyrosine bond between Tyr123 from GyrA and the cleaved DNA. The electron density for the four **t1** IPYs in the cleaved DNA in the 3.06 Å structure and the two **t3** IPYs in the 3.11 Å structure is shown in Supplementary Figure S2. The four **t1** IPYs all had reasonable density and were modeled in the same orientation, with the pendant aminopyrrolidine modeled in different orientations in the four **t1** IPYs (Supplementary Figure S2). In the 3.11 Å **t3** complex there was only one complex in the asymmetric unit. The electron density for one of the **t3** IPYs clearly showed it bound in the same orientation as the four **t1** IPYs, however, the second **t3** IPY had some density for its pyridine ring which suggested an alternative binding mode (Supplementary Figure S2H). In the final structure for the 3.11 Å **t3** complex, the compound at the second binding site has been modeled in two binding modes: the ‘standard’ IPY binding mode observed at the other five binding sites, and an ‘alternative’ binding mode. The electron density near this second **t3** IPY suggested that the DNA was partially cleaved and partially intact at this site (Supplementary Figure S2I). It may be that the two binding modes for the **t3** IPY at this second site are related to cleaved and re-ligated structures of the DNA. Some regions of the TOPRIM domains also had weak electron density in the IPY structures; most notably residues 438–445 of GyrB, a region close to the dimer interface.

## RESULTS

### Imidazopyrazinones (IPYs) stabilize DNA cleavage complexes

**Figure 1.**
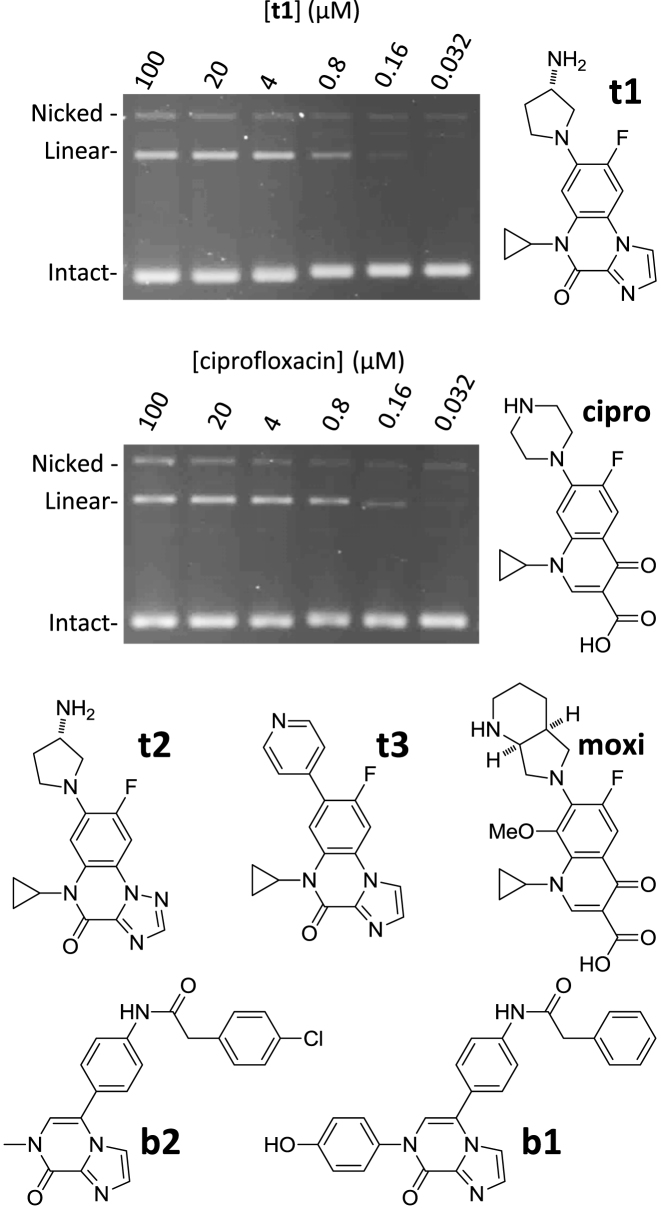
The imidopyrazinones (IPYs) stabilize gyrase–DNA cleavage complexes. The compounds used in this study are shown; **t1, t2** and **t3** are tricyclic IPYs; **b1** and **b2** are bicyclic IPYs. **t1** efficiently stabilizes double-strand cleavage complexes with *Escherichia coli* DNA gyrase and DNA (top) as evidenced by the induction of linear plasmid DNA in a cleavage assay (‘Materials and Methods’ section). Cleavage induced by ciprofloxacin (cipro) in the same assay is shown below. The migration positions of the different forms of the plasmid, Nicked, Linear and Intact are labeled on the left side. The enzyme concentration is identical for the two assays (around 250 ng of A_2_B_2_ tetramer for a 30 μl reaction). Compound concentrations are indicated in μM at the top of the lane. Moxifloxacin (moxi) is shown for reference as we use it for our structural discussion.

The IPY compounds have been shown to target bacterial type II DNA topoisomerases and show variable levels of activity against a panel of Gram-negative and/or Gram-positive bacteria (Jeannot *et al.* In revision in J Med Chem). Figure 1 shows structures of three tricyclic IPYs (**t1, t2** and **t3**) as well as two bicyclic IPYs (**b1** and **b2**) and the FQs ciprofloxacin and moxifloxacin. A DNA-cleavage assay performed with purified *Escherichia coli* DNA gyrase shows that the IPY **t1** induces levels of double-stranded cleavage comparable to those obtained with ciprofloxacin (Figure 1).

### IPYs bind at the quinolone pocket, in the same orientation as FQs, but without formation of a water–metal–ion bridge

**Figure 2.**
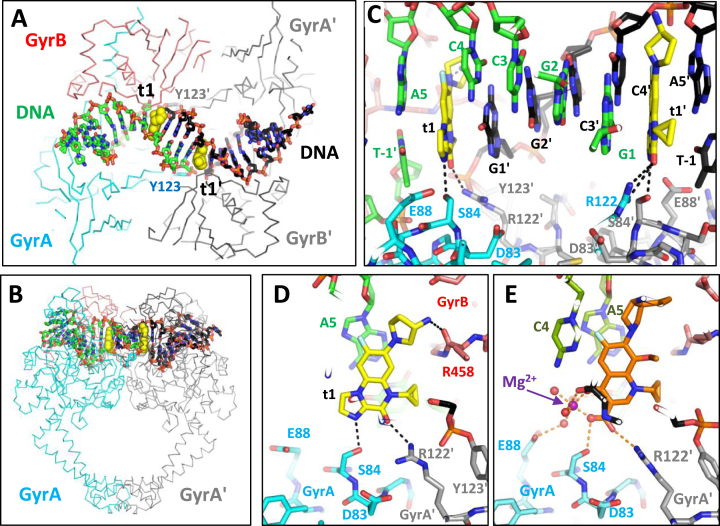
The 3.06 Å structure of **t1** with *Staphylococcus aureus* DNA gyrase and DNA. (**A**) View down the 2-fold axis of the 3.06 Å structure of **t1** with *S. aureus* DNA gyrase and DNA (one of two such complexes in the asymmetric unit). Note the 20 bp DNA duplex has been cleaved by tyrosines 123 (and 123’). (**B**) Orthogonal view of the complex with the 2-fold axis vertical. (**C**) Detailed view showing the two compounds (**t1** and **t1’**) bound at the two DNA cleavage sites. (**D**) **t1** binds in the DNA at the cleavage site, making interactions with *S. aureus* GyrA residues *Sa*S84, *Sa*R122 and the main-chain carbonyl of GyrB Arg 458. (**E**) An equivalent view of the 2.95 Å moxifloxacin complex showing the water–metal–ion bridge.

To gain insight into the poisoning mechanism of the IPYs, we solved crystal structures (Supplementary Table S1) of DNA–cleavage complexes formed between the *S. aureus* GyrB27-A56 (GKdel) fusion truncate (19,28,29) and a 200 bp DNA duplex (Supplementary Table S2) and compounds **t1** (Figures 1 and 2) and **t3** (Figure 1; Supplementary Figures S2 and 5). The 20 bp DNA duplex used in these crystal structures was very similar to that previously used in solving a 2.95 Å crystal structure with the FQ moxifloxacin (Supplementary Table S2 (29)). Although our program was aimed at developing new gyrase-targeted antibiotics active against Gram-negative pathogens (e.g. *E. coli*), crystallography of the *E. coli* gyrase–drug–DNA complexes is not straightforward and we therefore used the *S. aureus* DNA gyrase crystallography platform (28).

The structure reveals a high degree of similarity between the binding mode of **t1** and moxifloxacin involving hemi-intercalation of the compound at each DNA cleavage site (Figure 2C–E). Moxifloxacin (and other FQs) interacts with the protein via a water–metal–ion bridge (15–16,23,29) as shown in Figure 2E. The IPYs, such as **t1**, lack the metal-coordinating keto-acid groups of FQs and therefore cannot form a water–metal–ion bridge. Instead, **t1** contacts the protein through a direct hydrogen bond with *Sa*S84 on GyrA (equivalent to *EcS*83—the initial in italics denotes the species numbering for residues, *Ec: E. coli, Sa: S. aureus*), see Figure 2C and D.

The arginine positioned next to the catalytic tyrosine (R122) contacts the keto group of **t1/t3** (Figure 2C and D) in four of the six binding sites observed (four in the **t1** complex and two in the **t3** complex asymmetric units; Supplementary Figure S2). This arginine is conserved across type IIA topoisomerases and we found it to be essential for cleavage by the *S. aureus* fusion truncate (Supplementary Figure S3), but in the *S. aureus* gyrase crystal structures its position is quite variable (29). The distal nitrogen of the aminopyrrolidine group in **t1** has been modeled contacting the main-chain carbonyl of arginine 458 (Figure 2D) in some, but not all, subunits (Supplementary Figure S2). Substitution of this aminopyrrolidine group was part of the medicinal chemistry effort aimed at reducing cross-resistance with the FQs through improvement of the contact within the TOPRIM domain (e.g. compound **t3** where the aminopyrrolidine is replaced with a pyridine) although no breakthroughs were achieved (Jeannot *et al.* In revision in J med Chem).

### The IPYs poison DNA gyrase *in vitro* with partial FQ cross-resistance

The activity of tricyclic and bicyclic IPYs (Figure 1) was tested in a supercoiling inhibition assay (Table 1) on wild-type (wt) *E. coli* DNA gyrase and GyrA mutants *Ec*S83L (*Sa*S84), *Ec*D87A (*Sa*E88) and *Ec*D82N (*Sa*D83). These residues are located in the ‘Quinolone-Resistance-Determining-Region’, frequently found mutated in resistant clinical isolates (18,35–38). Based on the structures solved of FQs bound to DNA gyrase and topo IV, it has been established that both residues *Ec*S83 and *Ec*D87 are involved in a water–metal–ion bridge that is important for the stabilization of the ternary complex, explaining the resistance to FQs observed when these residues are mutated (15–16,23,29,39).

**Table 1.** Supercoiling IC_50_ values for various mutants and compounds (in μM)

	wild-type	*Ec*S83L	*Ec*D87A	*Ec*D82N
ciprofloxacin	0.45	8.7	3.2	4.7
**t1**	0.5	3.7	0.5	9.4
**t2**	1.7	9.5	1.7	4–20*
**t3**	1.6	12.0	1.6	4–20*
**b1**	2.6	1.0	ND	ND
**b2**	5.2	11.0	ND	ND

All these values were obtained with single preparations of wild type and mutant *E. coli* DNA gyrase; the GyrB subunit preparation was the same across all these measurements. Repeating these measurements with different preparations gave values that did not deviate by more than a factor 2. * The data could not be fitted so an approximate value was determined visually, i.e. the lower concentration of the indicated range gives more than 50% supercoiling and the higher concentration gives less. ND: Not Determined.

**Figure 3.**
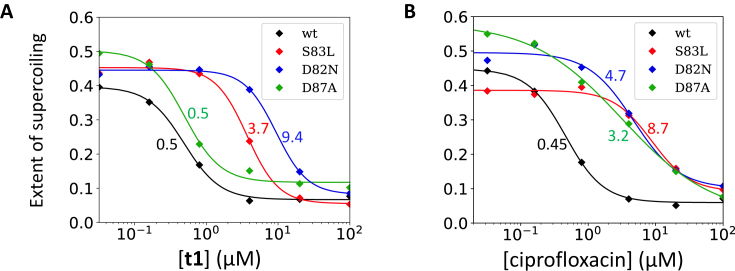
Inhibition of *Escherichia coli* DNA gyrase supercoiling by **t1** (**A**) and ciprofloxacin (**B**). The extent of supercoiling is defined as the proportion of supercoiled plasmid within the total material in the lane (supercoiling assay performed as described in ‘Materials and Methods’ section) and is plotted against compound concentrations (in μM). The diamonds are data points. The smooth curves are the best fit of a logistic function to the data points (‘Materials and Methods’ section). Ciprofloxacin (B) encounters resistance from all three mutants tested compared to the wt (curves are shifted to the right, see Table 1 for IC_50_ values). **t1** encounters resistance from *Ec*S83L and *Ec*D82N but not *Ec*D87A. The color-coded numbers next to the curves are the IC_50_ values (also reported in Table 1).

We found that the most active tricyclic IPY on ΔTolC *E. coli* (compound **t1**, Jeannot *et al.* In revision in J Med Chem) efficiently inhibited the negative supercoiling activity of *E. coli* DNA gyrase (IC_50_ = 0.5 μM, Figure 3 and Table 1). The S83L mutant enzyme showed resistance with an IC_50_ of 3.7 μM (around a 7-fold increase); in the same assay, ciprofloxacin displayed an ∼20-fold increase in IC_50_ with the *Ec*S83L mutant compared to the wt. The *Ec*D87A mutant enzyme did not show any resistance to the IPY compounds (IC_50_ of 0.49 μM, Table 1 and Figure 3). These results are consistent with the observed binding mode of **t1** in *S. aureus* DNA gyrase (Figure 2), in which **t1** makes a direct interaction with *Sa*S84 (*Ec*S83) while no direct contact is observed between the IPY and *Sa*E88 (*Ec*D87).

In the supercoiling inhibition assay the *Ec*D82N mutant showed cross-resistance to **t1** (IC_50_ of 9.4 μM). The *Ec*D82 (*Sa*D83) residue does not appear to be involved in binding to the IPYs or FQs at the cleavage site (Figure 2C–E) and likely has another role in poisoning (see below). Other tricyclic IPY compounds tested (**t2** and **t3**, Figure 1) similarly encountered partial resistance with the *Ec*S83L mutant, low to no resistance with the *Ec*D87A mutant, and high resistance with *Ec*D82N (a precise value could not be determined as we could not fit the data, but is likely to be between 4 and 20 μM) (Table 1). These results suggest a common binding mode across the tricyclic series. The stabilization of gyrase-induced double-stranded DNA cleavage stimulated by **t1** (Figure 1) was also observed with other IPYs (Table 2). The CC_50_ for **t1** was 0.75–0.98 μM (ciprofloxacin gave a CC_50_ of ∼0.2–0.42 μM in the same assay; Table 2 and Supplementary Figure S4). We have also performed CC_50_ measurements with ciprofloxacin and **t1** with all four versions of the enzyme (Supplementary Figure S4; wt, S83L, D87A, D82N) and the results largely mirror our IC_50_ measurement, i.e. for each compound the resistance profile obtained from IC_50_ values being similar to the one obtained from CC_50_ values.

**Table 2.** Cleavage CC_50_ values for various compounds on wild-type *E. coli* DNA gyrase

**Compound**	**CC_50_ (μM)**
ciprofloxacin	0.18
**t1**	0.75
**t2**	2.25
**t3**	1.08
**b1**	4.45

All these values were obtained with a single preparation of wild-type *E. coli* DNA gyrase. The CC_50_ value is defined as the concentration giving half the total amount of cleavage obtained at saturating amount of compound.

We have tested other compounds in the series for both cleavage and supercoiling inhibition, including two bicyclic IPYs (**b2** and **b1**, Figure 1), which had been briefly considered as initial starting points for an optimization program (Jeannot *et al.* In revision in J Med Chem). Like all the tricyclic IPYs tested, they proved able to inhibit supercoiling and stabilize cleavage by gyrase. The CC_50_ and IC_50_ values gave a good correlation across both bicyclic and tricyclic compounds (Tables 1 and 2) suggesting that the inhibition of supercoiling stems from the stabilization of gyrase–DNA cleavage complexes. The bicyclic IPY **b1** surprisingly displayed improved activity toward the *Ec*S83L mutant enzyme compared to the wt (Table 1). The difference is small but was reproduced with two independent preparations of both the mutant and wt enzyme. Interestingly the other bicyclic IPY, **b2**, was less active against *Ec*S83L, similar to the tricyclic series. Compound **b2** has a methyl group instead of a phenyl group attached to the pyrazinone core, suggesting that the small methyl group allows a binding mode similar to the tricyclic series, whereas encumbering it with the bigger phenyl group may change the binding mode, thereby altering the resistance profile.

### The cleaved state compared to an uncleaved binary enzyme–DNA complex

**Figure 4.**
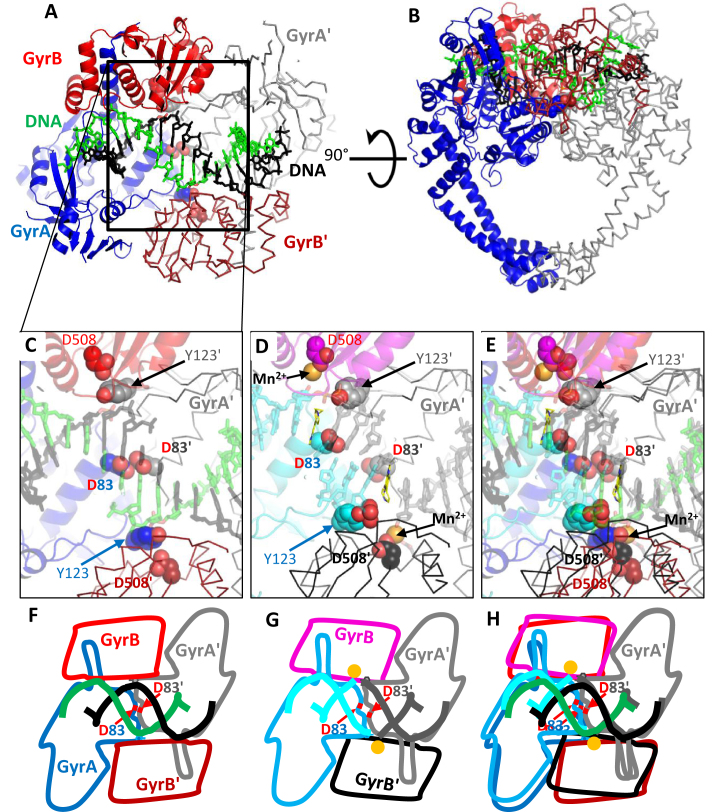
Comparison of the binary 2.65 Å complex of *Staphylococcus aureus* DNA gyrase and DNA, with the 3.11 Å **t3** complex. (**A** and **B**) Two orthogonal views of the 2.65 Å binary BA_BA' complex. The BA subunit is shown in cartoon representation, the BA' subunit in ribbon, the DNA is shown in stick representation. (**C**) Detailed view of the central region of the DNA in the binary complex, the central DNA is in A-DNA conformation. D508 from the TOPRIM domain does not bind a metal ion, presumably because it is too far away from the DNA for the metal ion also to interact with the DNA (cartoon and sticks are shown semi-transparent for clarity). Note that D83 and D83′ side-chains are in van der Waals contact at the twofold axis. (**D**) Equivalent view of the central region of the 3.11 Å structure of **t3** cleavage complex. (**E**) Superposition of D and E. (**F** and **G**) Schematics of domain orientations in the binary complex and the t3 complex. (**H**) Superposition of F and G.

To gain insight into the conformational transitions involved in the formation of a ternary cleavage complex, we solved the structure of a binary complex involving the same *S. aureus* gyrase core construct and DNA. Even though the overall conformation of this binary complex (Figure 4A and B) is quite similar to the IPY-poisoned ternary complex, this binary complex presents some clear differences from the IPY complexes and all other *S. aureus* complexes with DNA (19,29–30). In the IPY structures and all previously published *S. aureus* complexes with DNA, the WHD and TOPRIM domains within a subunit have been in approximately the same orientation ((29) and Supplementary Figure S5F). However, in the binary complex the TOPRIM domain is rotated by ∼12° around the Aα1 hinge (this hinge region is discussed elsewhere (21)), so that the metal-coordinating residues on the TOPRIM domain, including D508, have moved too far away from the DNA, preventing the metal ion from binding to the TOPRIM domain and also contacting the DNA (Figure 4C–H). In the binary complex, which is crystallized from the same crystallization conditions as many other *S. aureus* complexes, no metal ion is observed at the TOPRIM domain catalytic site.

Comparison of an IPY-poisoned ternary complex with the binary complex (Figure 4C–H; Supplementary Figures S5 and 6) shows a sliding motion of the two GyrA subunits past one another when transitioning from the binary to the ternary complex. The relative position of the two WHD domains in the binary complex resembles that seen in the apo *S. aureus* structure (PDB code: 2XCQ; (19)). In the binary complex the distance between the two scissile phosphates is 21.7 Å, and the DNA in this central region is in an A-DNA conformation (Supplementary Table S2). In contrast, in the 2.1 Å GSK299423 (an NBTI) structure with uncleaved DNA (PDB code: 2XCS; (19)), the distance between the two scissile phosphates is 25.7 Å, and metal ions are observed contacting the scissile phosphate (Supplementary Figures S5 and 6; (19)).

The *Sa*D83 residue (equivalent to *Ec*D82) is situated at the ‘sliding interface’ between the two GyrA subunits (Figure 4C–E). The two *Sa*D83 residues move apart when the complex transitions from the binary (unpoisoned) to the ternary (poisoned) conformation. Therefore, we surmise that mutation of this residue could influence the respective energetics of those two states and may have a net stabilizing effect on the binary complex. Modeling a *Sa*D83N mutation in the binary complex suggests that the unfavorable interaction between the two aspartic acid side-chains (*Sa*D83) observed in the binary complex can be replaced by two hydrogen bonds (not shown). The *Ec*D82N mutant is predicted to stabilize the uncleaved DNA conformation observed in the binary complex (and the apo structure) relative to cleaved conformations such as those observed in the IPY complexes (Figure 4).

### The *Ec*D82N mutation inhibits ATP-induced intrinsic DNA cleavage

Our structures reveal a degree of similarity between the binding mode of FQs and the tricyclic IPYs to DNA gyrase that is reflected by the cross-resistance with S83L. However, mutation of *Ec*D87, being involved in the binding of FQs and not **t1**, only confers resistance to FQs such as ciprofloxacin (Table 1). In contrast, the *Ec*D82N mutation confers resistance to both compounds, while not making direct contact with IPYs or FQs (Figure 2; *Sa*D83). We hypothesized that the *Ec*D82N mutation impairs the formation of a gyrase–DNA cleavage complex for both compounds by stabilizing the uncleaved conformation relative to the cleaved conformation rather than influencing compound binding *per se*.

**Figure 5.**
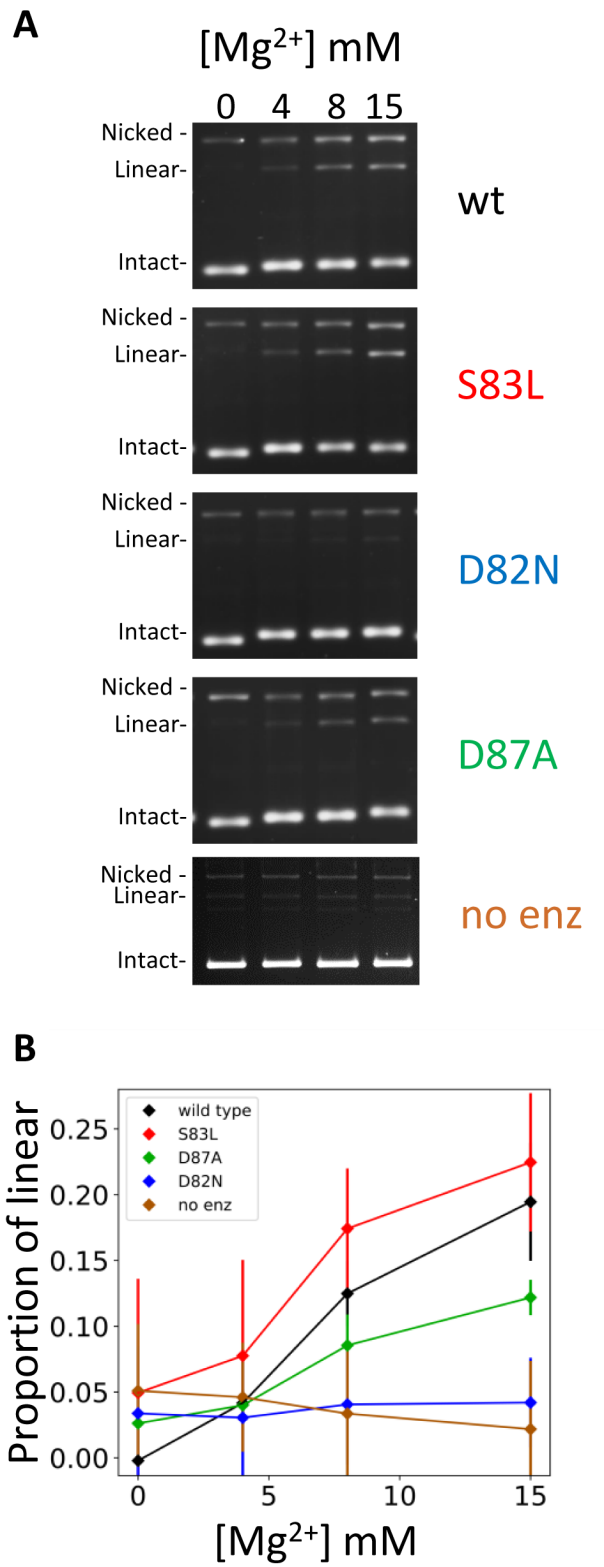
The D82N mutation affects the natural ability of DNA gyrase to cleave DNA with Mg^2+^ and ATP as co-factors. (**A**) Cleavage assays performed in the absence of compounds with wt, *Ec*S83L, *Ec*D82N and *Ec*D87A versions of the enzyme in the presence of ATP and either 0, 4, 8 or 15 mM MgCl_2_ added to the reaction. A no enzyme control (no enz) was also performed in the same conditions to assess the baseline of the assay. Mg^2+^ induces cleavage with all enzyme versions except *Ec*D82N. This cleavage is also dependent on ATP (not shown) for all versions of the enzyme. The slight migration shift of the intact band is due to the introduction of negative supercoiling which for all mutants and wt is dependent on ATP and Mg^2+^ (it is not observed in the no enzyme control). (**B**) Quantitation of the experiment in A. The average proportion of linear plasmid from three repeats for each point is plotted against MgCl_2_ concentration. The error bars are the standard deviation for the three repeats. For each enzyme versions, the amount of enzyme used was normalized at 2 μg per assay. This amount is higher used in Figure 1 (250 ng), 6 and 7 (400 ng) as cleavage is much lower in the absence of compound and therefore more enzyme is necessary to achieve detectable cleavage.

It is known that adenosine triphosphate (ATP) is required for multiple strand-passage events by gyrase. The catalytic cycle involves a transient cleaved intermediate presumably resembling the permanent cleavage complex stabilized by compounds. We tested the ability of ATP to induce cleavage on the wt, *Ec*S83L, *Ec*D82N and *Ec*D87A versions of the *E. coli* enzyme in the absence of any compounds (Figure 5). As expected, omission of ATP brings the cleavage level to nearly zero for all enzymes at any Mg^2+^ concentration (not shown). Likewise, in the absence of Mg^2+^ no cleavage is observed for all four enzymes, whereas increasing amounts of Mg^2+^ in the presence of ATP allows the detection of increasing amounts of DNA cleavage for all the mutants except with the *Ec*D82N enzyme, which fails to cleave DNA significantly in the presence of Mg^2+^ (Figure 5). We have been able to stabilize equivalent levels of cleavage between the D82N preparation and the other versions of the enzyme at saturating ciprofloxacin concentration (500 μM, Supplementary Figure S4). This shows that the D82N enzyme is still competent in utilizing Mg^2+^ to cleave DNA although it is much less efficient in stabilizing the cleaved state. This control rules out a higher incidence of inactive enzyme (i.e. misfolded) in the D82N preparation.

### Ciprofloxacin- and t1-induced cleavage complexes have a different Mg^2+^ dependency and similar kinetics of appearance

**Figure 6.**
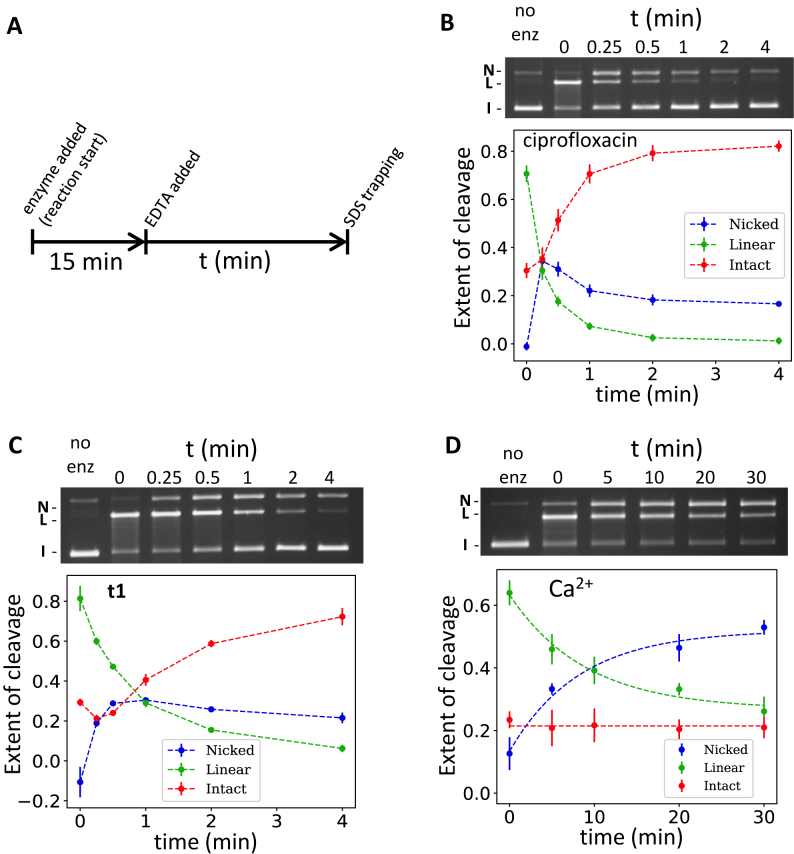
Sensitivity to EDTA of cleavage complexes stabilized by the IPYs, ciprofloxacin and Ca^2+^. (**A**) Outline of the experiment. The initial 15 min allows for the formation of cleavage complexes, which are then reversed by the addition of 8 mM EDTA (final). The SDS trapping is performed at various times after EDTA addition as for a normal cleavage assay (‘Materials and Methods’ section). One sample is kept free of enzyme for each of the three experiments and is trapped after the first 15 min. All three experiments are performed in the absence of ATP. (**B**) Reversion of ciprofloxacin cleavage complexes. An average of three repeats is plotted against the time spent in the presence of EDTA. The extent of cleavage is the proportion of each species to the total of the lane. Error bars are the standard deviation as in Figure 5. (**C**) Reversion of **t1** cleavage complexes, as in A and B. (**D**) Reversion of Ca^2+^-induced cleavage complexes as in A and B, but note that the time frame is much longer in this case. Approximately 400 ng of A_2_B_2_*Escherichia coli* wt gyrase was used in each assay.

The absence of the water–metal–ion bridge in the IPY structures prompted us to investigate the IPY cleavage complex’s sensitivity to the presence of Mg^2+^. We compared the kinetics of resealing in the presence of EDTA for both ciprofloxacin and **t1** (Figure 6B and C). We found that EDTA efficiently reseals ciprofloxacin-stabilized cleavage; the resealing occurred with a single-stranded cleaved intermediate (nicked). This nicked form appeared very quickly (within 15–30 s depending on the compound) in the resealing kinetics experiment, concomitant with the disappearance of linear DNA; conversely its disappearance was slow. The kinetics of resealing of cleavage stabilized by **t1** was very similar although the disappearance of the linear double-strand cleavage was slower, as was the appearance of the intact re-ligated form (Figure 6B and C). Consistent with this, the kinetics of appearance and disappearance of the nicked form was also slower with **t1** than with ciprofloxacin. The simplest explanation for these observations is that resealing occurs sequentially between the two cleavage sites. Re-ligation of one strand makes the linear form disappear and concomitantly produces the nicked form. The final resealing event might be slower or potentially affected by the EDTA treatment (see Discussion). Slower resealing of **t1**-induced cleavage by EDTA is consistent with the lack of a water–metal–ion bridge stabilizing the complex as opposed to the FQs.

It is interesting to see EDTA efficiently reversing the **t1**-stabilized cleavage, albeit at a reduced rate, despite the absence of a water-metal ion bridge. This suggests that the primary effect of EDTA during **t1**-induced cleavage reversion is to strip the metal ions from the catalytic cleavage site, thereby inducing resealing (whereas EDTA treatment presumably also destabilizes the water-metal bridge of the FQ ternary complex, thereby explaining the faster re-sealing kinetics). This would suggest that the catalytic-core metal is essential for cleavage maintenance.

Replacing Mg^2+^ by Ca^2+^ in the reaction has been shown to stabilize double-stranded DNA-cleavage by gyrase (24). However, unlike poisoning by the FQs, the enzyme is still able to introduce negative supercoils (which we confirmed in our experiments; not shown). Since Ca^2+^ ions can be chelated by EDTA (40), we performed the same experiment as above (Figure 6D) but with Ca^2+^ in place of Mg^2+^. The kinetics of resealing of Ca^2+^-induced cleavage is slower but strikingly similar to the profile displayed by FQs and IPYs. The disappearance of the linear DNA closely coincides with the appearance of the nicked DNA. We have independently fitted both datasets to single exponentials and the rate of linear disappearance was found to be very close to the rate of nicked appearance (0.11 and 0.12 min^−1^, respectively). Unlike the FQs and IPYs the amount of uncleaved DNA does not increase, showing that complete resealing does not occur.

**Figure 7.**
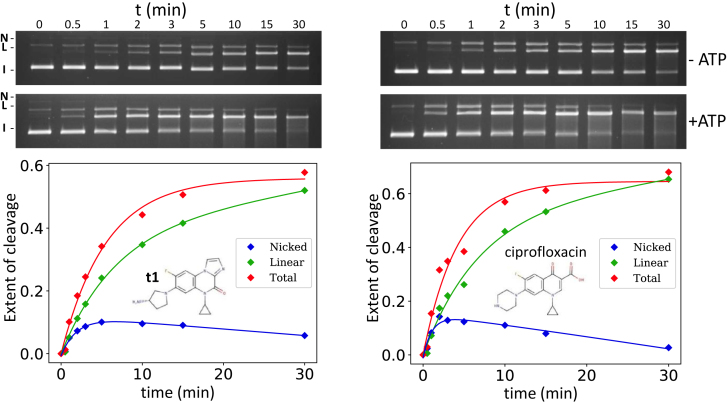
Kinetics of cleavage for ciprofloxacin and **t1**. The top panels show the results of cleavage assays with various times allowed for the formation of the cleavage complexes as indicated. The experiment was performed in the presence and the absence of ATP as indicated. The plot shows the quantitation for the minus ATP conditions only. The extent of cleavage is determined as in Figure 5. The data were fitted with a simple two-step process; it is indicative only. The single-strand intermediate and ATP stimulation of the kinetics of cleavage are apparent for both compounds. The rate of appearance is also quite similar for both compounds. Approximately 400 ng of A_2_B_2_*Escherichia coli* wt gyrase was used in each assay.

The single-strand cleaved form that we detected in the re-sealing experiments is reminiscent of the single-strand cleaved intermediate detected in the kinetics of appearance of ciprofloxacin-induced cleavage (41). In that study, ATP was shown to stimulate strand passage through the DNA gate by DNA gyrase. This result therefore suggests that the opening of the DNA gate greatly accelerates the formation of the cleavage complex. This is presumably due to ciprofloxacin not being able to directly trigger cleavage, but only to stabilize a cleaved conformation that is formed more readily when ATP hydrolysis is available to facilitate the partial opening of the DNA gate. To test whether the IPYs would behave similarly in terms of the kinetics of cleavage, we performed a cleavage assay in which the cleavage was initiated by adding the enzyme to a mixture of DNA substrate and the relevant compound (ciprofloxacin or **t1**) in the usual reaction buffer and transferred at 25°C. The cleavage was then trapped by SDS at various times. The reactions were carried out either in the presence or absence of ATP for both compounds. We found that in both cases the presence of ATP greatly stimulated the rate of appearance of the linear DNA (Figure 7). Moreover, the linear DNA appeared at a similar rate for ciprofloxacin and **t1**. We have quantified the product for the reaction carried out in the absence of ATP and found a similar rate for the appearance of linear DNA. In addition, a detectable nicked intermediate was observed for both compounds, again with similar rates (Figure 7). Our results are in line with previous results obtained for ciprofloxacin (41). This is consistent with an analogous binding and poisoning mechanism for the IPYs compared to the FQs.

## DISCUSSION

### The IPYs poison DNA gyrase by binding the quinolone pocket in a similar orientation to the one adopted by FQs

In this study, we have characterized a new class of non-quinolone bacterial topoisomerase inhibitors, the IPYs, and show that they efficiently inhibit the supercoiling activity of purified DNA gyrase due to their ability to stabilize a cleavage complex. This underlies their antibacterial activity as the cross resistance we observed *in vitro* is mirrored in whole cells with bacterial strains bearing FQ-resistance mutations in the gyrase genes (Jeannot *et al.* In revision in J Med Chem). Overall, we observed some correlation between the CC_50_/IC_50_ of compounds and their whole cell antibacterial activity. Taken together, this is consistent with a bactericidal action stemming from the stabilization of cleavage complexes, similar to the FQs. The orientation adopted by the tricyclic IPY **t1** was also similar to that adopted by FQs despite lacking the keto-carboxylic moiety that allows the FQ to directly contact DNA gyrase through a water-Mg^2+^ ion bridge. This is reminiscent of the binding mode of the quinazolinediones (42). Many structurally unrelated compounds are able to intercalate in the same position suggesting some flexibility in the ligand-binding pocket. This is consistent with a previous report suggesting two modes of binding for the FQs (43). Moreover, the DNA gate is a dynamic part of the molecule, being able to open to allow a DNA segment pass through, which might contribute to the flexibility in accommodating compounds. However, the IPYs are likely to establish some interactions with the DNA and/or protein that mirror those established by the FQs, allowing them to adopt a similar orientation. These interactions could be van der Waal forces and/or stacking interactions in addition to a direct interaction with the *Sa*S84 residue.

The structures of the tricyclic IPYs bound to the *S. aureus* core gyrase were supported by measuring the ability of the compounds to inhibit negative supercoiling by gyrase and key mutant forms of the enzyme. The tricyclic IPYs contact, via a hydrogen bond, *S. aureus* S84 (equivalent to *Ec*S83) but do not contact *Sa*D88 (*E*cD87) whereas the FQs establish a water–metal–ion bridge involving both residues (Figure 2D and E). Our experiments consistently showed that the *Ec*S83L and *Ec*D87A mutations both rendered gyrase resistant to inhibition by ciprofloxacin whereas the IPY compounds encountered resistance from *Ec*S83L but not *Ec*D87A. This was true for all three tricyclic IPYs tested. The case of the *Ec*D82 residue is more complex. It does not contact either the FQs or the IPYs but its mutation to an asparagine confers resistance to both at a high level. In contrast to *Ec*S83 and *Ec*D87 whose activity can be explained by their direct interaction with compounds, the D82N mutation seems to work by a different, ‘allosteric’ mechanism.

### The D82N mutation stabilizes uncleaved DNA suggesting a thermodynamic model for poisoning

The foregoing results suggest a distinction between residues whose mutation confers resistance to poisoning compounds. One class of residue (e.g. *Ec*S83 and *Ec*D87) would simply be involved in the binding and/or proper orientation of the compounds for poisoning: ‘direct’ mutations. The other class (e.g. *Ec*D82) would be generally involved in stabilizing the poisoned conformation or destabilizing the uncleaved conformation: ‘allosteric’ mutations. Our data show that, not only does the D82N mutant show resistance to IPYs and FQs (Figure 3 and Table 1), this mutation also inhibits the ATP-induced stabilization of both single- and double-cleaved DNA (Figure 5). In the 2.6 Å binary structure with uncleaved DNA, the equivalent *S. aureus* residue, D83, makes direct contact with its counterpart across the 2-fold axis (Figure 4). The mutation of the negatively charged aspartic acid side-chain (*Ec*D82/*Sa*D83) to an asparagine allows two hydrogen bonds to be made between the side-chains of Asn83, which we suggest will stabilize the binary complex with uncleaved DNA compared to the cleaved complex. If true, this would suggest that mutating this residue to an alanine, which is not able to form a similar hydrogen bond, would confer a lower level of resistance. This is indeed suggested by data reported previously (44), considering the low level of resistance of D82A mutant compared to the S83L mutant in MIC assay.

**Figure 8.**
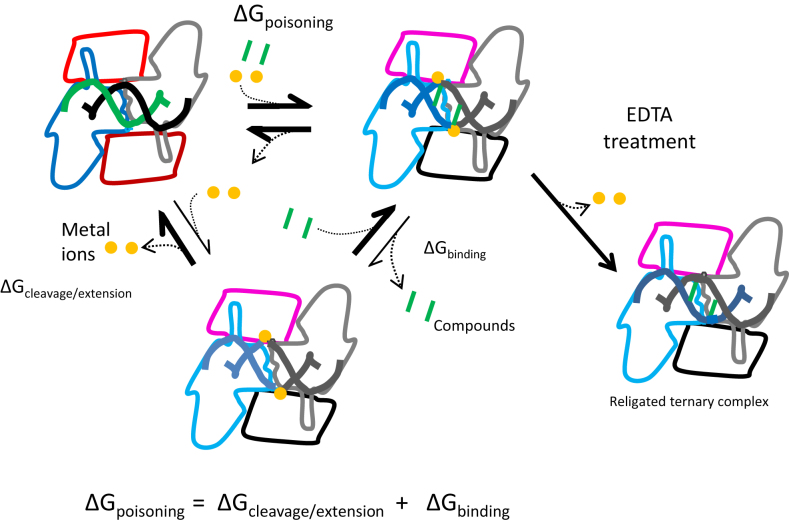
Thermodynamic model for the poisoning of DNA gyrase. Two factors that contribute to the free energy of poisoning can be distinguished. First, the conformational transition from an uncleaved complex to a cleaved complex, coupled with the sliding of the GyrA subunit, the tilting of the TOPRIM domain and the stretching of the DNA at the DNA gate. This favors metal-ion capture and therefore cleavage. This transition is presumably not energetically favorable and constitutes a ‘prelude’ to the full opening of the DNA gate for strand passage. Second, the binding of the compound to this general poisoned conformation which stabilizes it through its favorable binding energy. EDTA addition strips the metal from the poisoned conformation, which results in re-sealing. In the case of ciprofloxacin it presumably also destabilizes the ternary complex, resulting in a transition toward the binary complex and the catalytic Mg^2+^ being expelled at a higher rate. This is consistent with our observation of a higher re-sealing rate by EDTA for the ciprofloxacin cleavage complexes as compared with **t1**.

In the binary crystal structure (Figure 4) the central 4 bp of DNA between the two active sites are in an A-DNA-like conformation and the distance between the scissile phosphates is 21.7 Å (Supplementary Figure S7). In contrast, two previous crystal structures of bacterial type IIA topoisomerase with uncleaved DNA showed the central 4 bp of DNA stretched between the two catalytic sites so that the distance between the two scissile phosphates is 25.7 or 25.5 Å (Supplementary Figure S7) (19,42). It is presumably not thermodynamically favorable for the DNA gate to adopt this partially opened conformation as it extends the DNA into a non-A conformation and reduces the extent of interaction at the protein interface (Figure 4). Therefore, for a compound to successfully intercalate and poison the enzyme, the binding energy afforded through direct contact would have to compensate for the unfavorable opening of the DNA gate. Within that view, any factor that influences the intrinsic dynamics of the DNA gate (i.e. the free energy of this transition between closed and partially opened) would also influence its ability to be poisoned (Figure 8). The partial opening of the DNA gate is achieved by a conformational change involving a tilting of the TOPRIM domain and is likely coupled to divalent metal capture, which allows DNA cleavage. Consistent with this hypothesis, our binary complex structure does not involve any metal at the catalytic cleavage sites.

The intercalation of compounds and partial opening of the DNA gate is also coupled with a sliding of the two GyrA subunits relative to one another. (Structural transitions involving sliding of interfaces in type II topoisomerases has also been suggested in work on human topoisomerase IIβ (45,46).) Within the binary complex of the *S. aureus* gyrase core, the *Ec*D82 residue from each side of the dimer point toward each other at the dyad axis. The *Ec*D82N mutation reduces the ability of the enzyme to cleave DNA in the presence of ATP, without any compound present. This suggests that the *Ec*D82N mutation disfavors the cleaved state even when no intercalating compound is present. We surmise that the structural change observed between the binary and ternary complexes resembles the one that the enzyme undergoes during its normal strand-passage activity, stimulated by ATP. Interestingly, the GyrA sliding interface constitutes the binding pocket of the novel bacterial type II topoisomerase inhibitors (NBTIs) (19), again consistent with this sliding motion being involved in transition to the cleaved state. Moreover, mutation of the *Ec*D82 residue in *E. coli* and the equivalent residue in *Pseudomonas aeruginosa* can confer resistance to the NBTIs (47). This could be due to the direct interaction of the residue with the NBTI; however it is tempting to speculate that *Ec*D82’s intrinsic function in cleavage also plays a role.

Other residues whose mutation confers resistance have been characterized that do not contact the poisoning compound in any obvious manner. For instance, in work by Aldred *et al.* (48), a *Bacillus anthracis* topo IV mutant confers resistance to both FQs and quinazolinediones and is likely not involved in directly contacting either. We surmise that this is a case analogous to the *Ec*D82N mutation. Moreover a *Schizosaccharomyces pombe* topo II mutant has been shown to display intrinsically higher DNA cleavage activity in the presence of ATP (and absence of compound) (49) and confers upon yeast cells hypersensitivity to doxorubicin (50) and TOP-53 (an etoposide derivative; Germe, T. and Cooper, J.P., unpublished data), both of which are able to poison topo II by intercalation at the cleavage site, much like the FQs. Although a direct interaction of both structurally unrelated compounds with this residue in yeast topo II cannot be ruled out (however unlikely), this suggests a mirror case of *Ec*D82N and furthermore suggests that mutations that affect the intrinsic ability to cleave DNA also affect poisoning in all type IIA topoisomerases.

Presumably, ATP hydrolysis favors the structural transition that we call ‘partial opening’ as a pre-requisite for strand passage. This is consistent with the observation that ATP favors Mg^2+^-dependent cleavage by the unpoisoned enzyme (Figure 5). We have also shown that ATP accelerates the poisoning of gyrase by both ciprofloxacin and **t1** (Figure 7), which is consistent with the dynamics of the enzyme being intrinsically involved in the efficiency of poisoning.

The kinetics of appearance of cleavage complexes were strikingly similar between ciprofloxacin and **t1**, occurring at a comparable rate and with a detectable single-strand broken intermediate, as reported previously for ciprofloxacin (41). This work also suggested that ciprofloxacin binding occurred quickly, indeed faster than the rate of cleavage appearance, and it was also reported that cleavage is not necessary for the binding of ciprofloxacin to the enzyme–DNA complex (51). It is therefore likely that the kinetics of cleavage reflect the rate at which the enzyme can reach the cleaved state rather than the rate of binding of the compound. We hypothesize that the IPY series also binds quickly to the enzyme, the similar kinetics reflecting a similar path to the cleaved state, through a single-strand broken intermediate.

### The role of the metal at the cleavage site

We compared the EDTA-induced re-sealing dynamics of the cleavage complexes stabilized by either ciprofloxacin or **t1**. The addition of EDTA results in the re-sealing of double-stranded cleavage complexes stabilized by **t1** as well as those stabilized by ciprofloxacin (Figure 6). Since no water-Mg^2+^ bridge is involved in the binding of **t1**, the effect of EDTA is most likely to remove the Mg^2+^ from the cleavage site, destabilizing the cleavage competent conformations of the enzyme (Figure 8). Our data suggest that Mg^2+^ is essential for the maintenance of the cleaved state for both compounds (Figure 6). In addition, the faster re-sealing kinetics for ciprofloxacin is consistent with the hypothesis that re-sealing could occur both by stripping the compound from the enzyme (through the destabilization of the water–metal–ion bridge) and also by stripping the metal from the catalytic sites. In the case of **t1**, since no metal is involved in binding, the compound would presumably remain bound to the enzyme, unaffected by EDTA, re-sealing occurring only through stripping of the metal from the catalytic sites and therefore with slower kinetics. The tricyclic IPYs are therefore reminiscent of the quinazolinediones, which also show a reduced sensitivity for Mg^2+^ due to the absence of a water–metal–ion bridge and, like the IPYs, bypass some FQ-resistance mutations (39,52–53).

The re-ligation also involves a single-strand intermediate for both **t1** and ciprofloxacin (Figure 6). This nicking is not observed with the enzyme alone at this concentration and it is transient, although slow to disappear completely. The ability of gyrase to stabilize single-stranded cleavage complexes has been observed before with the thiophene compounds and NBTIs (19,21). It has also been observed that ATP induces a detectable single-stranded cleavage complex with the unpoisoned enzyme (i.e. without compounds) (21). The single-strand cleaved state, which constitutes an intermediate during re-ligation, is stabilized by both **t1** and ciprofloxacin. Interestingly when we investigated re-ligation of cleavage induced by Ca^2+^ we observed similar results (Figure 6D). It should be noted that Ca^2+^ can sustain negative supercoiling, albeit at a reduced rate (our unpublished data). Addition of EDTA to remove the Ca^2+^ also results in resealing on one side, converting double-cleaved to singly-cleaved DNA (Figure 6D). However, unlike ciprofloxacin and **t1**, the single-strand cleavage observed after EDTA treatment of Ca^2+^-stabilized cleavage complexes persists. This suggests an asymmetry between the two metal-binding catalytic sites, and that the first and second DNA-re-ligation steps are different, at least in the presence of calcium.

Nicking induced by EDTA has been observed by other investigators (52,53). However, it remains unclear how this nicking arises. We observed that EDTA also fails to re-seal the nicking observed with the unpoisoned enzyme in the presence of ATP (data not shown and (21)). We propose that the enzyme can naturally reach a single-strand cleaved state after which resealing can be affected by EDTA. Moreover, we believe this state is an obligatory intermediate during re-ligation.

It has been proposed that type IIA topoisomerases catalyze cleavage through a two-metal mechanism with two coordination sites for metal ions (normally Mg^2+^ although it is replaced by Mn^2+^ in the *S. aureus* gyrase crystal structures) (54–57). The A-site coordinates a metal ion that can directly catalyze the transesterification reaction, whereas the B-site contacts the next, non-scissile phosphate via a water molecule (Supplementary Figure S6) and is thought to have a ‘DNA-docking’ function for cleavage. Across the array of gyrase core structures in which the DNA is cleaved by the enzyme, a metal has generally been observed at the B-site (3′). Only with an uncleaved ternary complex (with the NBTIs) has the metal been observed at the A-site (where it presumably directly catalyses cleavage and/or re-ligation) (19). It should be noted that cleavage and re-ligation do not necessarily occur identically. For instance, the ‘docking’ function of the B-site is presumably superfluous for re-ligation, when the DNA is already cleaved and held in proximity by the phospho-tyrosine bond. At the A-site, the metal contacts the scissile phosphate (unlike the B-site) and it therefore is reasonable that this coordination site would change upon cleavage and DNA gate opening, resulting in the metal leaving the A-site but not the B-site, explaining the lack of metal in the A-site only in structures of gyrase bound to already cleaved DNA. Therefore, re-ligation would would need to recruit metal ion from the solution or, we speculate, the B-site, since the docking function is no longer required after cleavage.

The observation that EDTA treatment results in resealing on one side only suggests that stripping the metal from one site results in resealing, whereas the metal from the other site is not stripped at the same rate. If the propensity of the metal to be pulled from the B-site to the A-site is affected by the structural asymmetry of the cleavage complex, one could imagine that on the side where this motion is hindered, the metal could be stripped without going through the A-site, thereby producing an ‘un-re-sealable’ single-stranded cleavage complex, consistent with our observations. If the motion is unhindered, the metal is readily able to go through the A-site before being stripped and therefore the net result would be resealing. This is consistent with the observation that higher EDTA concentrations produce higher levels of nicking (52). A high concentration of EDTA would favor stripping the metal from the B-site before it had time to move back to the A-site, thereby producing an un-re-sealable complex with no metal complexed to cleavage site.

This metal motion would therefore be coupled to the conformational transition to a binary complex (namely the tilting of the TOPRIM domain away from the cleavage site on DNA). This is consistent with our observation of a binary complex devoid of metal at the cleavage site. It is also consistent with the observation of fast re-sealing kinetics of ciprofloxacin–gyrase–DNA cleavage complexes. EDTA-mediated destabilization of the ternary complex (due to destabilization of the water–metal–ion bridge) favors transition to the binary complex, which in turn favors the metal ion moving to the A-site, catalyzing re-ligation and leaving the enzyme. We further speculate that the transition to the binary complex state occurs sequentially (one side of the dyad axis after the other) thereby explaining our observation of a single-strand intermediate.

Asymmetry between the two sides has been observed with structures of etoposide gyrase–DNA cleavage complexes (29). Etoposide stabilizes a mixture of single- and double-strand breaks and we observed that this single-strand cleavage cannot be re-sealed by EDTA, correlating a structural asymmetry of the cleavage complex with an asymmetry in EDTA sensitivity (unpublished data). This is consistent with the relative motion of the TOPRIM domains and WHD domains, which control DNA extension, also control the status of the metal at the cleavage site.

### A possible alternative orientation for the binding of bicyclic IPY in the same pocket

The tricyclic IPY compounds adopt an orientation that still favors contact with *EcS*83 and results in cross-resistance with quinolones. One bicyclic IPY (**b1**) did not display cross-resistance with the S83L mutant but rather showed an increased activity against that mutant; the S83L mutant was resistant to the other bicyclic tested, much like the tricyclic compounds. The differences between the two bicyclic IPYs are a chlorine substituent on the top aromatic ring for **b2** that is not present for **b1** (Figure 1) and the pyrazinone core is substituted on N1 by a methyl for **b2** and by a much bulkier phenol group for **b1**. It is the protruding imidazo nitrogen atom of the IPY moiety that establishes contact with S83 in the case of the tricyclic compounds. The simpler interpretation is therefore that the same atom establishes contact with S83 in the case of **b2** explaining the resistance data. Compound **b1** would itself adopt a different conformation within the pocket that is favored by the absence of S83. One possibility could be that the phenolic group on **b1** prevents the IPY from establishing contact with S83, forcing the compound to adopt a different orientation. It would be interesting to determine this putative alternative orientation as it could open new avenues for the development of new poisons. Although the bicyclic IPYs present a possible avenue to develop new intercalative poisons for antibiotic purposes, their low activity against the enzyme and lack of significant antibacterial activity would have to be overcome and there is currently no obvious route for this optimization.

### Summary and general conclusion

In conclusion, the IPYs have been shown to bind to the FQ pocket of gyrase in a fashion similar to the FQs despite the exchange of the 4-oxo and carboxylic acid groups of FQs for a fused imidazole moiety in IPYs. These IPYs were shown to have partial cross-resistance with the FQs which led to the termination of their optimization. This study also points toward a general poisoning mechanism whereby the partial opening of the DNA gate, involving lateral sliding of the two GyrA subunits against each other and tilting of the TOPRIM domains toward the dyad axis, favors Mg^2+^ capture and DNA cleavage. Compounds poison the enzyme by impeding the closure of the DNA gate, their binding/intercalation energy compensating for the energetic cost of keeping the DNA stretched, and the DNA gate partially opened, thus favoring a conformation where DNA can readily be cleaved, catalyzed by Mg^2+^. This opening of the DNA gate could involve some asymmetry causing the two cleavage sites to not be competent for re-ligation at the same time. Our data suggest a model in which this is due to differing ability in mobilizing the metal from the B-site (where it is seen in crystal structures) to the A-site (where it catalyses the re-sealing reaction), although this is only a speculative model and our data do not contradict the two-metal model proposed for cleavage. In addition, our results with the bicyclic IPYs also suggest a degree of flexibility in the orientation of compounds within the binding pocket, which could have implications for medicinal chemistry efforts in the development of new bacterial topoisomerase II poisons that are able to circumvent target-mediated resistance to FQ.

## Supplementary Material

Supplementary Data
