# A new class of antibacterials, the imidazopyrazinones, reveal structural transitions involved in DNA gyrase poisoning and mechanisms of resistance

**DOI:** 10.1093/nar/gky241

**Published:** 2018-03-27

**Authors:** Thomas Germe, Judit Vörös, Frederic Jeannot, Thomas Taillier, Robert A Stavenger, Eric Bacqué, Anthony Maxwell, Benjamin D Bax

**Affiliations:** 1 Department of Biological Chemistry, John Innes Centre, Norwich Research Park, Norwich NR4 7UH, UK; 2 Sanofi R&D, TSU Infectious Diseases, 1541 Avenue Marcel Mérieux, 69280 Marcy L’Etoile, France; 3 Antibacterial Discovery Performance Unit, Infectious Diseases Therapy Area Unit, GlaxoSmithKline, 1250 Collegeville Road, Collegeville, PA 19426, USA; 4 Platform Technology and Science, GlaxoSmithKline, Medicines Research Centre, Gunnels Wood, Road, Stevenage, Hertfordshire SG1 2NY, UK

*Nucleic Acids Research* (2018), gky181, https://doi.org/10.1093/nar/gky181

The authors would like to point out that the DNA substrate used in the enzyme reactions in this paper was plasmid pNO1 (1), not pBR322 as stated in the paper. These two plasmids are very similar and this error has no impact on the results and conclusions of the paper.
