# Supplementary material for: A new class of antibacterials, the imidazopyrazinones, reveal structural transitions involved in DNA gyrase poisoning and mechanisms of resistance
Source: Nucleic Acids Res. 2018 Mar 10;46(8):4114–28. doi: 10.1093/nar/gky181 (PMC5934680; doi:10.1093/nar/gky181)
Supplement: Supplementary Data [file gky181_supp.zip › IPYII_Suppl_REVISIONS_WithCorrections3.docx]

**Supplementary for:**

**A new class of antibacterials, the imidazopyrazinones, reveal structural transitions involved in DNA gyrase poisoning and mechanisms of resistance.**

Thomas Germe^1^, Judit Voros^1^, Frederic Jeannot^2^, Thomas Tailler^2^, Robert A Stavenger^3^, Eric Bacqué^2^, Anthony Maxwell^1*^, Benjamin D Bax^1,4^

^1^Dept. Biological Chemistry, John Innes Centre, Norwich Research Park, Norwich NR4 7UH, UK; ^2^Sanofi R&D, TSU Infectious Diseases, 1541 Avenue Marcel Mérieux, 69280, Marcy L’Etoile, France; ^3^Antibacterial Discovery Performance Unit, Infectious Diseases Therapy Area Unit, GlaxoSmithKline, 1250 Collegeville Rd., Collegeville, Pennsylvania, PA19426, USA; ^4^Platform Technology and Science, GlaxoSmithKline, Medicines Research Centre, Gunnels Wood Road, Stevenage, Hertfordshire, SG1 2NY, UK;

Supplementary materials and methods

Synthesis and characterization of compound b1

Compound 1 was synthesised as outlined in Scheme S1. General Methods: The nomenclature of the compounds is based on ACDLABS software, version 11.01. All chemicals and anhydrous solvents were obtained from commercial suppliers and used without further purification. Reactions were monitored using thin layer chromatography (TLC), visualisation was realized under UV light (254 nm). Purification of title compounds was accomplished by flash column chromatography using silica gel 60 (eluents are given in parentheses). NMR spectra were recorded on a BRUKER DPX 300MHz spectrometer operating at a proton frequency of 300.133 MHz. ^1^H-chemical shifts were referenced to the DMSO-d_6_ signals (^1^H: 2.50 ppm) and CDCl_3_ (1H: 7.26 ppm). Electron impact mass spectrometry was performed on an Finnigan SSQ700 mass. The final compound was characterized using the following instruments and conditions: The ^1^H and ^13^C NMR spectra were performed, in d_6_-DMSO, at 303K, on a Bruker Avance I operating at a proton frequency of 600 MHz and a ^13^C-carbon frequency of 151 MHz. Chemical shifts (δ, expressed in ppm) were referenced to the solvent signals (2.50 ppm and 39.52 ppm). Coupling constants (J) are given in Hertz. High-resolution mass spectrometry was performed on an LCT Premier XE Waters TOF spectrometer (mode electrospray ES+) in combination with a Waters Acquity UPLC. Following the purification step, solid compounds (intermediate and targeted compounds) were generally not crystallized and used/characterized as such.

**III**

**VI**

**V**

**IV**

**II**

**I**

Scheme 1: Synthesis of **VI**. Conditions: a) Acetone, K_2_CO_3_, reflux. b) HC(NMe_2_)_2_O*t*Bu (Bredereck’s reagent), toluene, reflux. c) p-benzyloxyaniline, acetic acid, reflux. d) TiCl_3_, acetic acid, water, r.t. e) phenylacetyl chloride, triethylamine, chloroform. f) Pd/C, DMF.

Ethyl 1-[(4-nitrophenyl)methyl]-1H-imidazole-2-carboxylate (**I**):

To a refluxed mixture of potassium carbonate (31 g, 224 mmol) and ethyl 1H-imidazole-2-carboxylate (19.6 g, 139 mmol) in acetone (400 ml) was added dropwise 1-(bromomethyl)-4-nitrobenzene (30.2 g, 139 mmol) diluted in acetone (100ml). Heating was pursued for 2 h and the reaction mixture was filtered over celite, the filtrate was concentrated under reduced pressure. Diisopropyl ether (500 ml) was added and evaporation was pursued until 200 ml of solvent remained. Diisopropyl ether (300 ml) was added to the residue. After overnight trituration, the solid was filtered in order to obtain the title compound (37 g, 96%) as a pink solid.

**I**

^1^H NMR (300 MHz, CDCl_3_): δ 8.21 (d, 2 H), 7.3 (m, 3H), 7.17 (s, 1H), 5.77 (s, 2H), 4.39 (q, 2H), 1.4 (t, 3H). EI MS m/z: 275.

Ethyl 1-[(E)-2-(dimethylamino)-1-(4-nitrophenyl)ethenyl]-1H-imidazole-2-carboxylate (**II**):

To a solution of ethyl 1-[(4-nitrophenyl)methyl]-1H-imidazole-2-carboxylate (xx) (37 g, 134 mmol) in toluene (450 ml) was added Bredereck’s reagent (41.6 ml, 201 mmol). The reaction mixture was refluxed for 6 hours then Bredereck’s reagent was added (5 mL, 24 mmol), heating was pursued for another 12 h. After concentration under reduced pressure, the residue was taken up in diisopropyl ether (400 mL) and ethanol (40 mL), filtered, and washed successively with diisopropyl ether and pentane to afford the expected compound (20.2 g, 45%) as an orange solid.

**II**

^1^H NMR (300 MHz, DMSO-d_6_): δ 8.02 (d, 2H), 7.54 (s, 1H), 7.48 (s, 1H), 7.35 (s, 1H), 6.88 (d, 2H), 4.2 (q, 2H), 2.7 (s, 6H), 1.18 (t, 3H).

7-[4-(benzyloxy)phenyl]-5-(4-nitrophenyl)imidazo[1,2-a]pyrazin-8(7H)-one (**III**):

To a solution of ethyl 1-[(E)-2-(dimethylamino)-1-(4-nitrophenyl)ethenyl]-1H-imidazole-2-carboxylate (3 g, 9 mmol) in acetic acid (70 ml) was added 4-(benzyloxy)aniline (1.97 g, 9.9 mmol). The resulting mixture was refluxed overnight then filtered. The solid was washed with ethanol and diisopropyl ether to afford the title compound as a yellow solid (3.4 g, 86%).

**III**

^1^H NMR (300 MHz, DMSO-d_6_): δ 8.39 (d, 2H), 7.98 (d, 2H), 7.82 (s, 1H), 7.62 (s, 1H), 7.58-7.3 (m, 8H), 7.19 (d, 2H), 5.19 (s, 2H).

5-(4-aminophenyl)-7-[4-(benzyloxy)phenyl]imidazo[1,2-a]pyrazin-8(7H)-one (**IV**):

To a solution of 7-[4-(benzyloxy)phenyl]-5-(4-nitrophenyl)imidazo[1,2-a]pyrazin-8(7H)-one (xx) (3.3 g, 7.5 mmol) in acetic acid (20 mL) and water (20 ml) was added dropwise titanium(III) chloride (60 mL, 7 mmol) while keeping the temperature below 30°C. The reaction mixture was stirred for 4 hours at room temperature and filtered. The filtrate was concentrated under reduced pressure and the resulting solid was taken up in diisopropyl ether. The resulting precipitate was filtered and dried in order to obtain the expected compound as a beige solid (2.26 g, 74%).

**IV**

^1^H NMR (300 MHz, DMSO-d_6_): δ 7.7-7.3 (m, 12H), 7.18 (d, 1H), 6.95 (s, 1H), 6.7 (d, 2H), 5.55 (s, 2H); 5.2 (s, 2H). EI MS m/z: 408.

N-(4-{7-[4-(benzyloxy)phenyl]-8-oxo-7,8-dihydroimidazo[1,2-a]pyrazin-5-yl}phenyl)-2-phenylacetamide (**V**):

To a solution of triethylamine (0.36 ml, 2.6 mmol) and 5-(4-aminophenyl)-7-[4-(benzyloxy)phenyl]imidazo[1,2-a]pyrazin-8(7H)-one (1 g, 2.4 mmol) in chloroform (15 ml). Phenylacetyl chloride (0.35 ml, 2.6 mmol) diluted in chloroform (15 ml) was then added dropwise and stirring was pursued overnight at room temperature. The reaction mixture was then concentrated under reduced pressure and the crude product was submitted to a silica gel column chromatography, using methanol / dichloromethane (5/95) as eluent, followed by trituration in diisopropyl ether and filtration. The solid was taken up in chloroform (40 mL), the resulting organic phase was washed successively with acetic acid (2 x 50 ml), a solution of saturated sodium hydrogen carbonate (2 x 25 ml), dried , filtered and evaporated in order to obtain the title compound as a yellow solid (1.2 g, 95%).

**V**

^1^H NMR (300 MHz, DMSO-d_6_): δ 10.35 (s, 1H), 7.78-7.0 (m, 21H), 5.11 (s, 2H), .3.6 (s, 2H).

N-{4-[7-(4-hydroxyphenyl)-8-oxo-7,8-dihydroimidazo[1,2-a]pyrazin-5-yl]phenyl}-2-phenylacetamide (**VI**):

In an RBF were charged DMF (50 ml), Pd/C (0.12 g, 1.1 mmol), and N-(4-{7-[4-(benzyloxy)phenyl]-8-oxo-7,8-dihydroimidazo[1,2-a]pyrazin-5-yl}phenyl)-2-phenylacetamide (1.2 g, 2.2 mmol). The resulting mixture was stirred under hydrogen atmosphere until completion. The mixture was filtered and the filtrate concentrated under reduced pressure. The residue was purified via a silica gel column chromatography, using methanol / dichloromethane (5/95) as eluent. After concentration under reduced pressure of the appropriate fractions the resulting solid was taken up in diisopropyl ether and pentane to afford the expected compound as white solid (0.65 g, 68%).

**VI**

^1^H NMR (600 MHz, DMSO) δ 10.38 (s, 1H), 9.74 (s, 1H), 7.76 (d, J = 8.75 Hz, 2H), 7.68 (d, J = 1.17 Hz, 1H), 7.59 (d, J = 8.75 Hz, 2H), 7.55 (d, J = 1.17 Hz, 1H), 7.38 – 7.20 (m, 7H), 7.04 (s, 1H), 6.87 (d, J = 8.84 Hz, 2H), 3.67 (s, 2H).

^13^C NMR (151 MHz, DMSO) δ 169.88, 157.61, 152.63, 140.60, 137.52, 136.29, 133.31, 131.39, 129.73, 129.55, 129.54, 128.80, 128.55, 127.05, 125.42, 120.30, 119.76, 119.22, 116.45, 115.92, 43.84. HRMS (ESI-TOF) m/z: calcd for C_26_H_20_N_4_O_3_ [M+H]^+^, 437,1614; found, 437,1610.

**Table S1.** X-ray data collection and refinement statistics.

|  | **t1** (cleaved 20-448T-U DNA) [PDB code: 6FQM] | **t3** (cleaved 20-448T-U DNA) [PDB code: 6FQS] | Binary complex (uncleaved 20-447T DNA) [PDB code: 6FQV] |
| --- | --- | --- | --- |
| **Data collection** |  |  |  |
| Beamline | DIAMOND – i03 | ESRF id23eh1 | ESRF ID29 |
| Space group | P2_1_ | P6_1_ | P2_1_ |
| Cell dimensions a,b,c (Å)  α, β, γ (°) | 88.3, 171.7, 124.9  90.0, 102.9, 90.0 | 94.0, 94.0, 420.5  90.0, 90.0, 120.0 | 93.3, 124.7, 155.2  90.0, 95.7, 90.0 |
| Wavelength (Å) | 0.97625 | 0.97600 | 0.9762 |
| Resolution range (Å) | 60-3.06 (3.14-3.06) | 48.34-3.11 (3.22-3.11) | 20.0-2.6 (2.64-2.60) |
| No. of unique reflections | 67466 (4994) | 37639 (3666) | 107487 (5291) |
| Multiplicity | 3.4 (3.4) | 10.5 (9.7) | 4.5 (4.6) |
| Completeness (%) | 98.6 (99.9) | 99.9 (99.1) | 99.1 (99.3) |
| R_merge_ (%) | 8.2 (63.7) | 19.4 (157.2) | 10.2 (102.7) |
| I/σI | 8.3 (1.6) | 9.5 (1.5) | 9.3 (1.3) |
| CC (1/2) | 0.994 (0.654) | 0.995 (0.532) | 0.994 (0.527) |
| **Refinement** |  |  |  |
| Resolution (Å) | 3.06 (3.14-3.06) | 3.11 (3.19-3.11) | 20.0-2.60 (2.67-2.60) |
| No. reflections | 64116(4977) | 37524 (2865) | 107487 (7934) |
| *R*_work/_ *R*_free_ (%) | 19.5/23.5 (35.5/37.5) | 18.0/21.0 (28.9/33.5) | 20.0/23.6 (24.1/26.2) |
| No. Atoms | 21111 | 11808 | 23192 |
| Protein | 19283 | 10722 | 21067 |
| DNA | 1585 | 870 | 1567 |
| Ligand/ion | 188 | 104 | 88 |
| Water | 55 | 112 | 470 |
| B-factors |  |  |  |
| Protein | 75 | 89 | 63 |
| DNA | 59 | 94 | 67 |
| Ligand/ion | 84 | 120 | 82 |
| Water | 86 | 86 | 52 |
| R.m.s deviations |  |  |  |
| Bond lengths (Å) | 0.013 | 0.011 | 0.009 |
| Bond angles (º) | 1.68 | 1.34 | 1.03 |

**Table S2.** DNA sequences in two crystal structures with IPYs and a binary complex.

DNAs are self-complementary and form 20 bp homoduplexes. The same DNA, 20-448T-U, was used for crystal structures with **t1**A and **t3**A. This DNA was cleaved by the enzyme forming a covalent bond with Tyr123 from GyrA (indicated by a ^Y^). The nucleotide sequence of 20-448-T, used in the 2.95-Å structure with moxifloxacin (pdb code: 5cdq), is also shown, with nucleotides differing from 20-448T-U highlighted in red. The 20-447T DNA which was used in the binary structure was not cleaved in the binary complex, nucleotides differing from 20-448T are highlighted in red. Below the 20-447T DNA, the stretches of DNA that adopt A-DNA or B-DNA conformation are given – as assigned by the program 3DNA (1,2). Note the terminal bases are not complementary.

| POSITION 5'-3'  3'-5' | -8 -7 -6 -5 -4 -3 -2 -1 1 2 3 4 5 6 7 8 9 10 11 12  12 11 10 9 8 7 6 5 4 3 2 1 -1 -2 -3 -4 -5 -6 -7 -8 |
| --- | --- |
| 20-448T-U 5'-3'  3'-5' | **G A G A G T A T  ^Y^G G C C A T A C U C T T**  **T T C U C A T A C C G G^Y^  T A T G A G A G** |
| 20-448T 5'-3'  3'-5' | **G A G C G T A T  ^Y^G G C C A T A C G C T T**  **T T C G C A T A C C G G^Y^  T A T G C G A G** |
| 20-447T 5'-3'  3'-5'  3DNA – type | **G A G C G T A C G G C C G T A C G C T T**  **T T C G C A T G C C G G C A T G C G A G**  B B A A A A A A A B B |

By convention topo IIA DNA sequences are numbered relative to the cleavage sites (between -1 and 1).

**Supplementary references**

1. Lu, X.J. and Olson, W.K. (2003) 3DNA: a software package for the analysis, rebuilding and visualization of three-dimensional nucleic acid structures. *Nucleic Acids Res*, **31**, 5108-5121.

2. Lu, X.J. and Olson, W.K. (2008) 3DNA: a versatile, integrated software system for the analysis, rebuilding and visualization of three-dimensional nucleic-acid structures. *Nat Protoc*, **3**, 1213-1227.
