# Supplementary material for: A new class of antibacterials, the imidazopyrazinones, reveal structural transitions involved in DNA gyrase poisoning and mechanisms of resistance
Source: Nucleic Acids Res. 2018 Mar 10;46(8):4114–28. doi: 10.1093/nar/gky181 (PMC5934680; doi:10.1093/nar/gky181)
Supplement: Supplementary Data [file gky181_supp.zip › IPYs_SUPPLEMENTARY_figures_REVISIONS_WithCorrections3.pptx]

## Slide 1
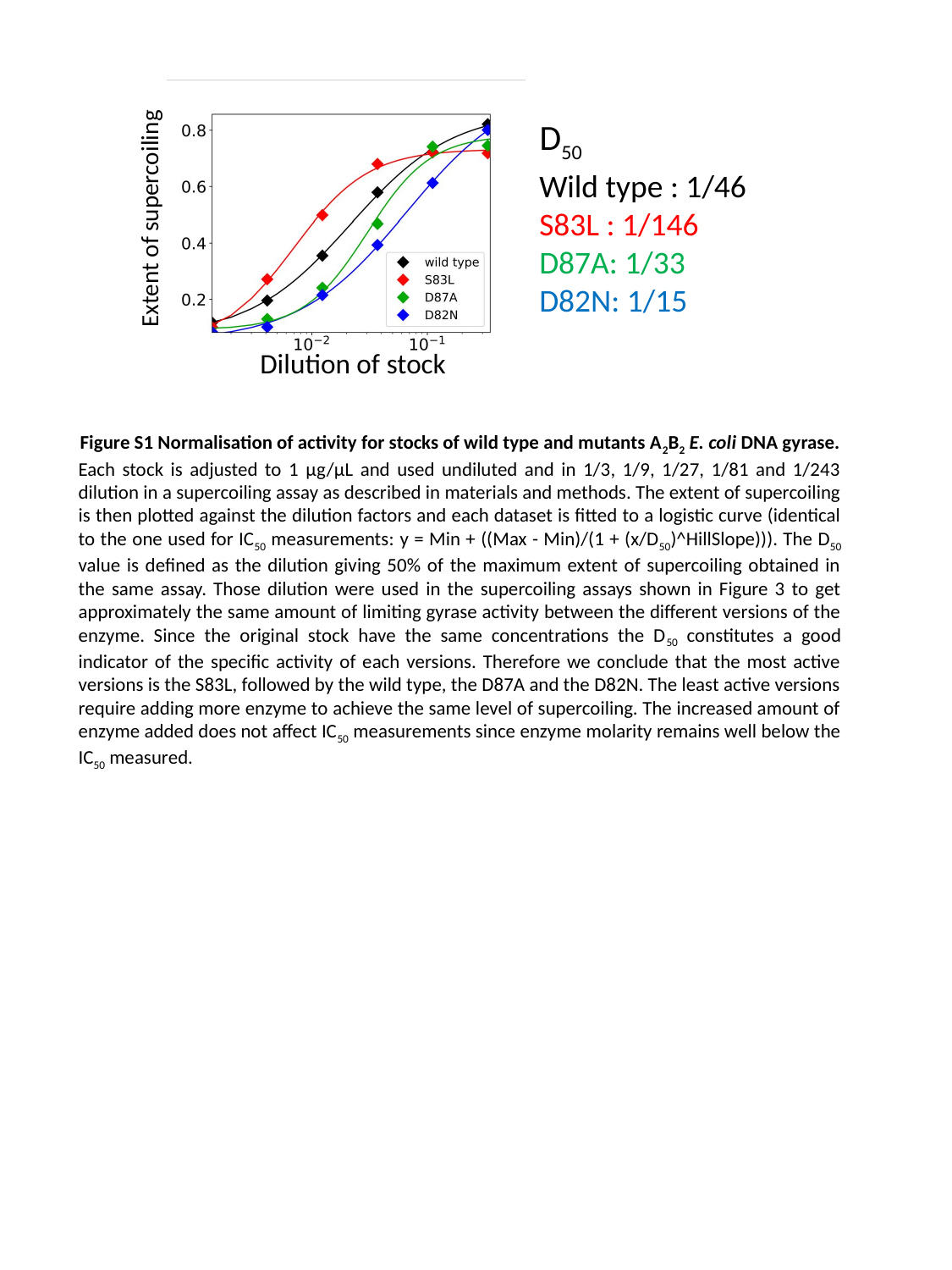

D50
Wild type : 1/46
S83L : 1/146
D87A: 1/33
D82N: 1/15
Extent of supercoiling
Dilution of stock
Figure S1 Normalisation of activity for stocks of wild type and mutants A2B2 E. coli DNA gyrase.
Each stock is adjusted to 1 µg/µL and used undiluted and in 1/3, 1/9, 1/27, 1/81 and 1/243 dilution in a supercoiling assay as described in materials and methods. The extent of supercoiling is then plotted against the dilution factors and each dataset is fitted to a logistic curve (identical to the one used for IC50 measurements: y = Min + ((Max - Min)/(1 + (x/D50)^HillSlope))). The D50 value is defined as the dilution giving 50% of the maximum extent of supercoiling obtained in the same assay. Those dilution were used in the supercoiling assays shown in Figure 3 to get approximately the same amount of limiting gyrase activity between the different versions of the enzyme. Since the original stock have the same concentrations the D50 constitutes a good indicator of the specific activity of each versions. Therefore we conclude that the most active versions is the S83L, followed by the wild type, the D87A and the D82N. The least active versions require adding more enzyme to achieve the same level of supercoiling. The increased amount of enzyme added does not affect IC50 measurements since enzyme molarity remains well below the IC50 measured.

## Slide 2
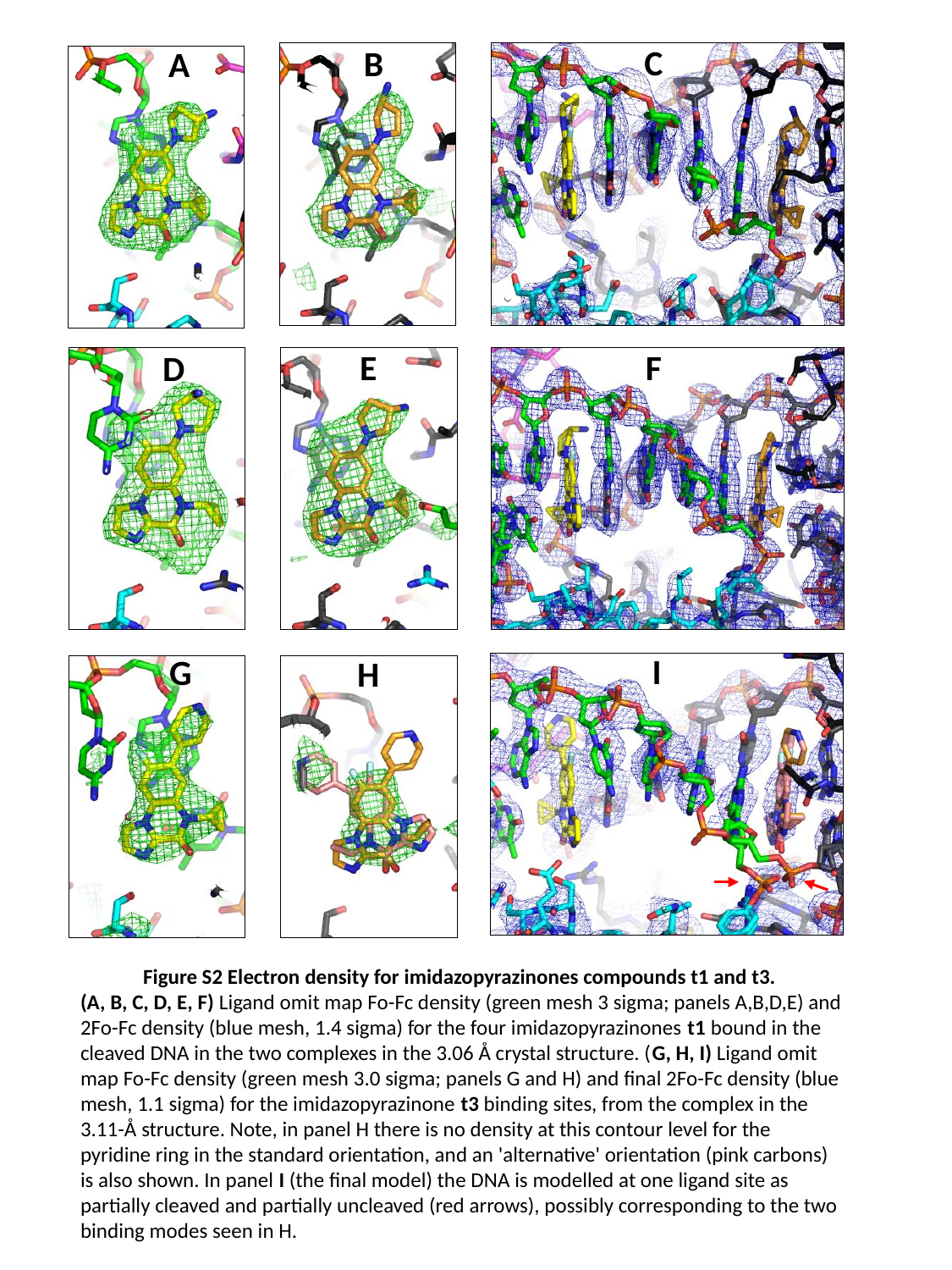

C
B
A
F
E
D
I
G
H
Figure S2 Electron density for imidazopyrazinones compounds t1 and t3.
(A, B, C, D, E, F) Ligand omit map Fo-Fc density (green mesh 3 sigma; panels A,B,D,E) and 2Fo-Fc density (blue mesh, 1.4 sigma) for the four imidazopyrazinones t1 bound in the cleaved DNA in the two complexes in the 3.06 Å crystal structure. (G, H, I) Ligand omit map Fo-Fc density (green mesh 3.0 sigma; panels G and H) and final 2Fo-Fc density (blue mesh, 1.1 sigma) for the imidazopyrazinone t3 binding sites, from the complex in the 3.11-Å structure. Note, in panel H there is no density at this contour level for the pyridine ring in the standard orientation, and an 'alternative' orientation (pink carbons) is also shown. In panel I (the final model) the DNA is modelled at one ligand site as partially cleaved and partially uncleaved (red arrows), possibly corresponding to the two binding modes seen in H.

## Slide 3
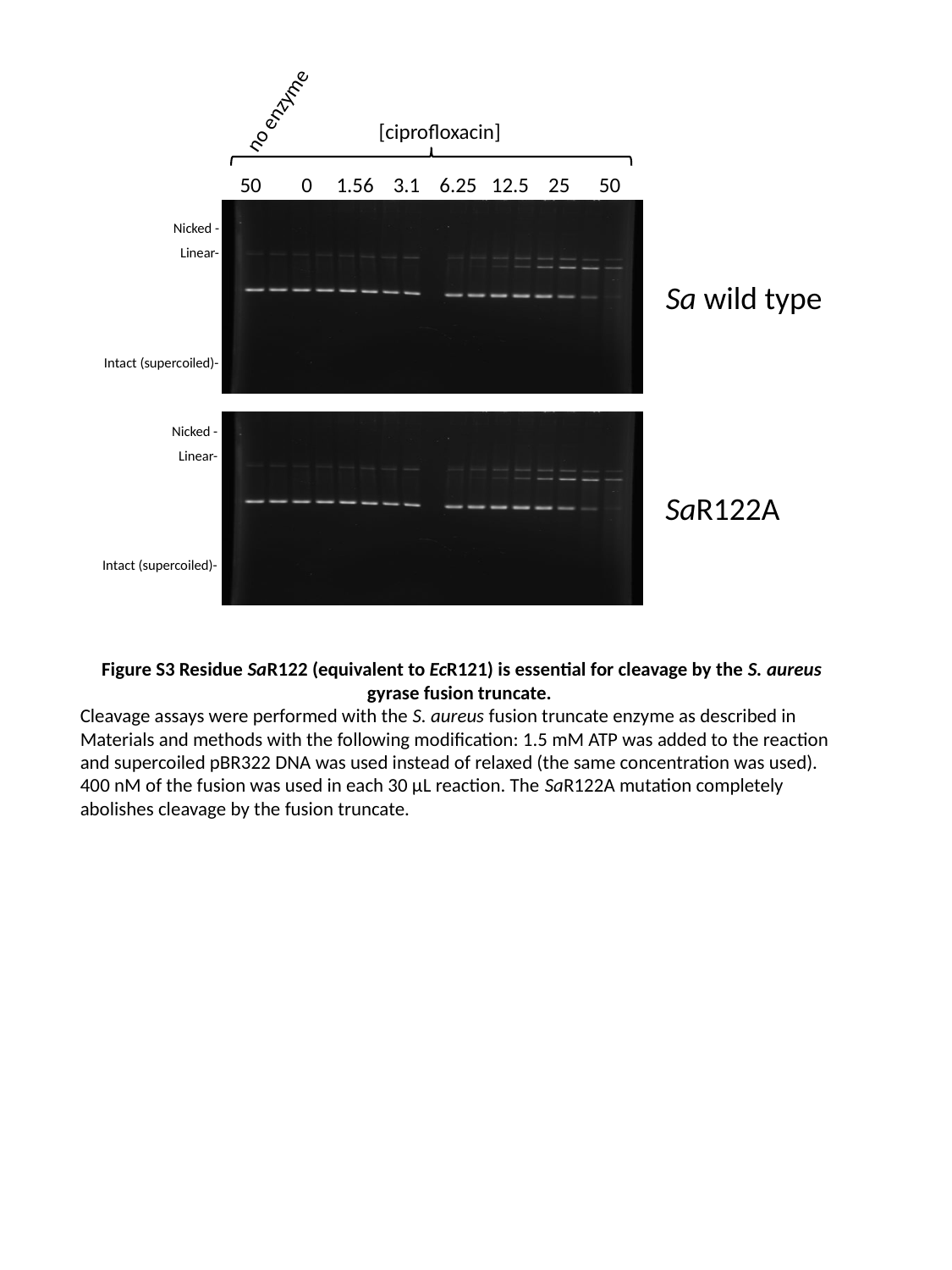

no enzyme
[ciprofloxacin]
50
0 1.56 3.1 6.25 12.5 25 50
Nicked -
Linear-
Sa wild type
Intact (supercoiled)-
Nicked -
Linear-
SaR122A
Intact (supercoiled)-
Figure S3 Residue SaR122 (equivalent to EcR121) is essential for cleavage by the S. aureus gyrase fusion truncate.
Cleavage assays were performed with the S. aureus fusion truncate enzyme as described in Materials and methods with the following modification: 1.5 mM ATP was added to the reaction and supercoiled pBR322 DNA was used instead of relaxed (the same concentration was used). 400 nM of the fusion was used in each 30 µL reaction. The SaR122A mutation completely abolishes cleavage by the fusion truncate.

## Slide 4
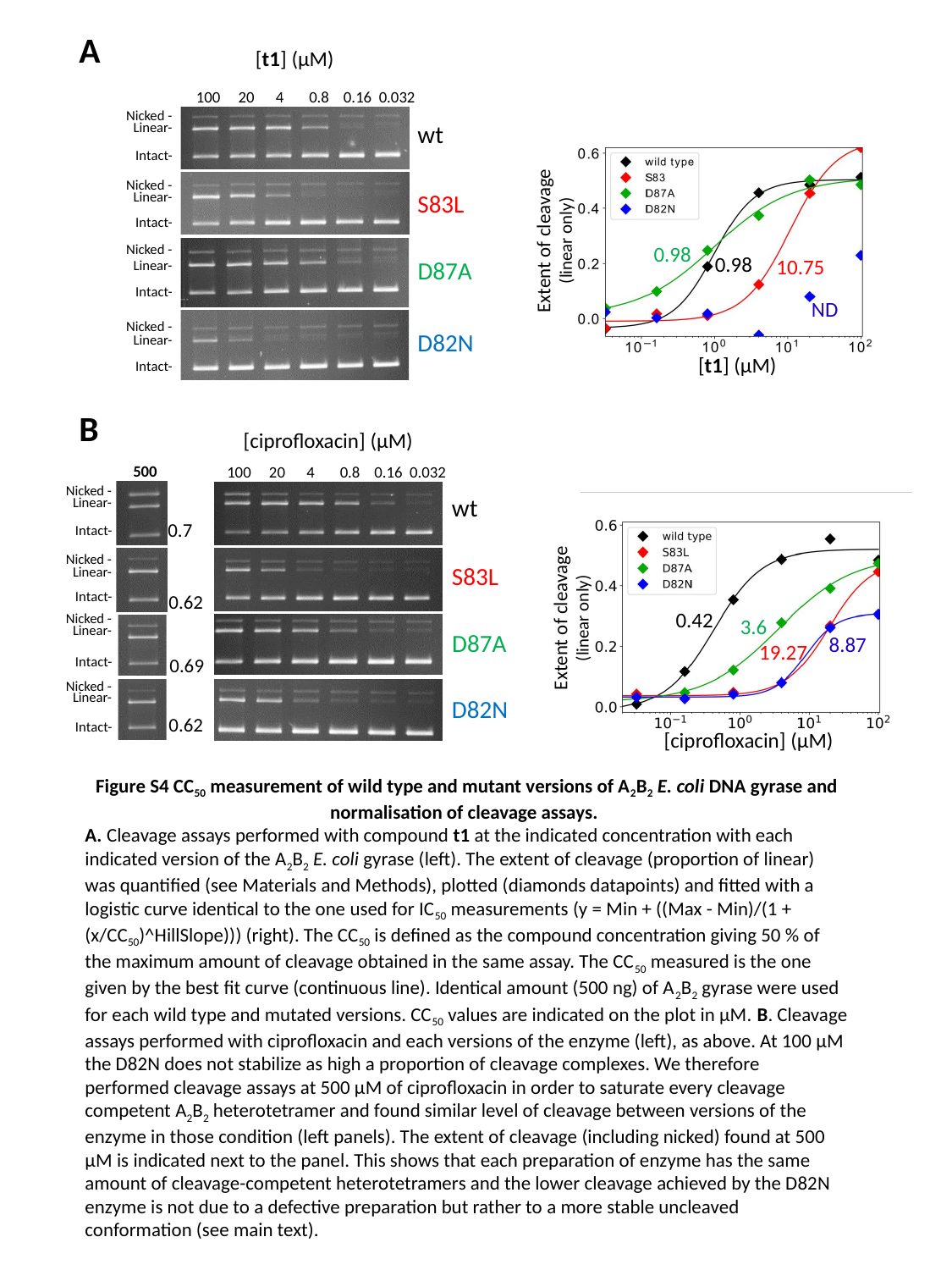

A
[t1] (µM)
100 20 4 0.8 0.16 0.032
Nicked -
Linear-
wt
Intact-
Nicked -
Linear-
S83L
Intact-
Extent of cleavage
(linear only)
Nicked -
0.98
0.98
10.75
D87A
Linear-
Intact-
ND
Nicked -
D82N
Linear-
[t1] (µM)
Intact-
B
[ciprofloxacin] (µM)
500
100 20 4 0.8 0.16 0.032
Nicked -
wt
Linear-
0.7
Intact-
Nicked -
S83L
Linear-
Intact-
0.62
Extent of cleavage
(linear only)
0.42
Nicked -
3.6
Linear-
D87A
8.87
19.27
Intact-
0.69
Nicked -
Linear-
D82N
0.62
Intact-
[ciprofloxacin] (µM)
Figure S4 CC50 measurement of wild type and mutant versions of A2B2 E. coli DNA gyrase and normalisation of cleavage assays.
A. Cleavage assays performed with compound t1 at the indicated concentration with each indicated version of the A2B2 E. coli gyrase (left). The extent of cleavage (proportion of linear) was quantified (see Materials and Methods), plotted (diamonds datapoints) and fitted with a logistic curve identical to the one used for IC50 measurements (y = Min + ((Max - Min)/(1 + (x/CC50)^HillSlope))) (right). The CC50 is defined as the compound concentration giving 50 % of the maximum amount of cleavage obtained in the same assay. The CC50 measured is the one given by the best fit curve (continuous line). Identical amount (500 ng) of A2B2 gyrase were used for each wild type and mutated versions. CC50 values are indicated on the plot in µM. B. Cleavage assays performed with ciprofloxacin and each versions of the enzyme (left), as above. At 100 µM the D82N does not stabilize as high a proportion of cleavage complexes. We therefore performed cleavage assays at 500 µM of ciprofloxacin in order to saturate every cleavage competent A2B2 heterotetramer and found similar level of cleavage between versions of the enzyme in those condition (left panels). The extent of cleavage (including nicked) found at 500 µM is indicated next to the panel. This shows that each preparation of enzyme has the same amount of cleavage-competent heterotetramers and the lower cleavage achieved by the D82N enzyme is not due to a defective preparation but rather to a more stable uncleaved conformation (see main text).

## Slide 5
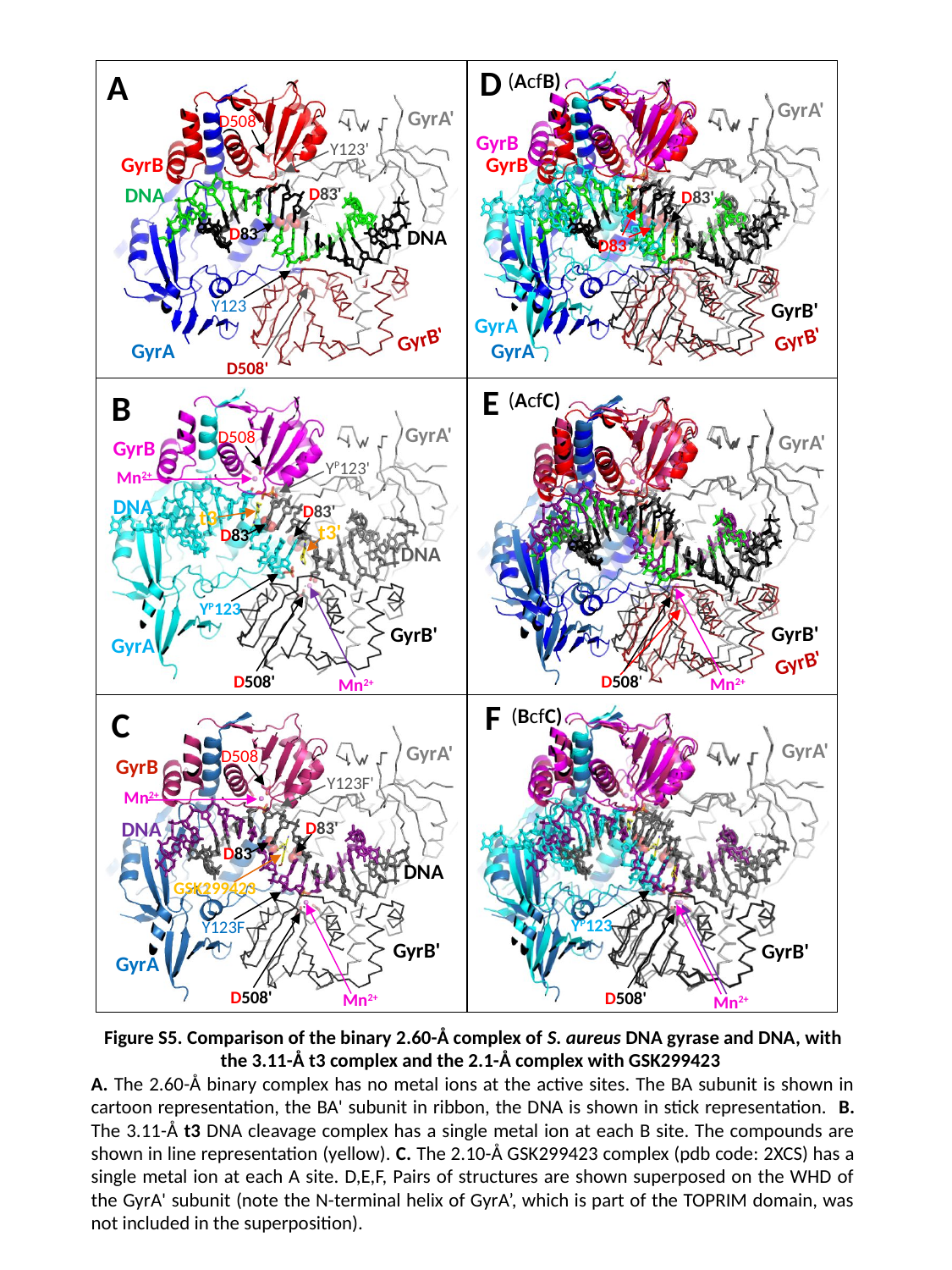

D
A
(AcfB)
GyrA'
GyrA'
D508
GyrB
Y123'
GyrB
GyrB
DNA
D83'
D83'
D83
DNA
D83
Y123
GyrB'
GyrA
GyrB'
GyrB'
GyrA
GyrA
D508'
E
B
(AcfC)
GyrA'
D508
GyrA'
GyrB
YP123'
Mn2+
DNA
D83'
t3
t3'
D83
DNA
YP123
GyrB'
GyrB'
GyrA
GyrB'
D508'
D508'
Mn2+
Mn2+
F
(BcfC)
C
GyrA'
GyrA'
D508
GyrB
Y123F'
Mn2+
DNA
D83'
D83
DNA
GSK299423
YP123
Y123F
GyrB'
GyrB'
GyrA
D508'
D508'
Mn2+
Mn2+
Figure S5. Comparison of the binary 2.60-Å complex of S. aureus DNA gyrase and DNA, with the 3.11-Å t3 complex and the 2.1-Å complex with GSK299423
A. The 2.60-Å binary complex has no metal ions at the active sites. The BA subunit is shown in cartoon representation, the BA' subunit in ribbon, the DNA is shown in stick representation. B. The 3.11-Å t3 DNA cleavage complex has a single metal ion at each B site. The compounds are shown in line representation (yellow). C. The 2.10-Å GSK299423 complex (pdb code: 2XCS) has a single metal ion at each A site. D,E,F, Pairs of structures are shown superposed on the WHD of the GyrA' subunit (note the N-terminal helix of GyrA’, which is part of the TOPRIM domain, was not included in the superposition).

## Slide 6
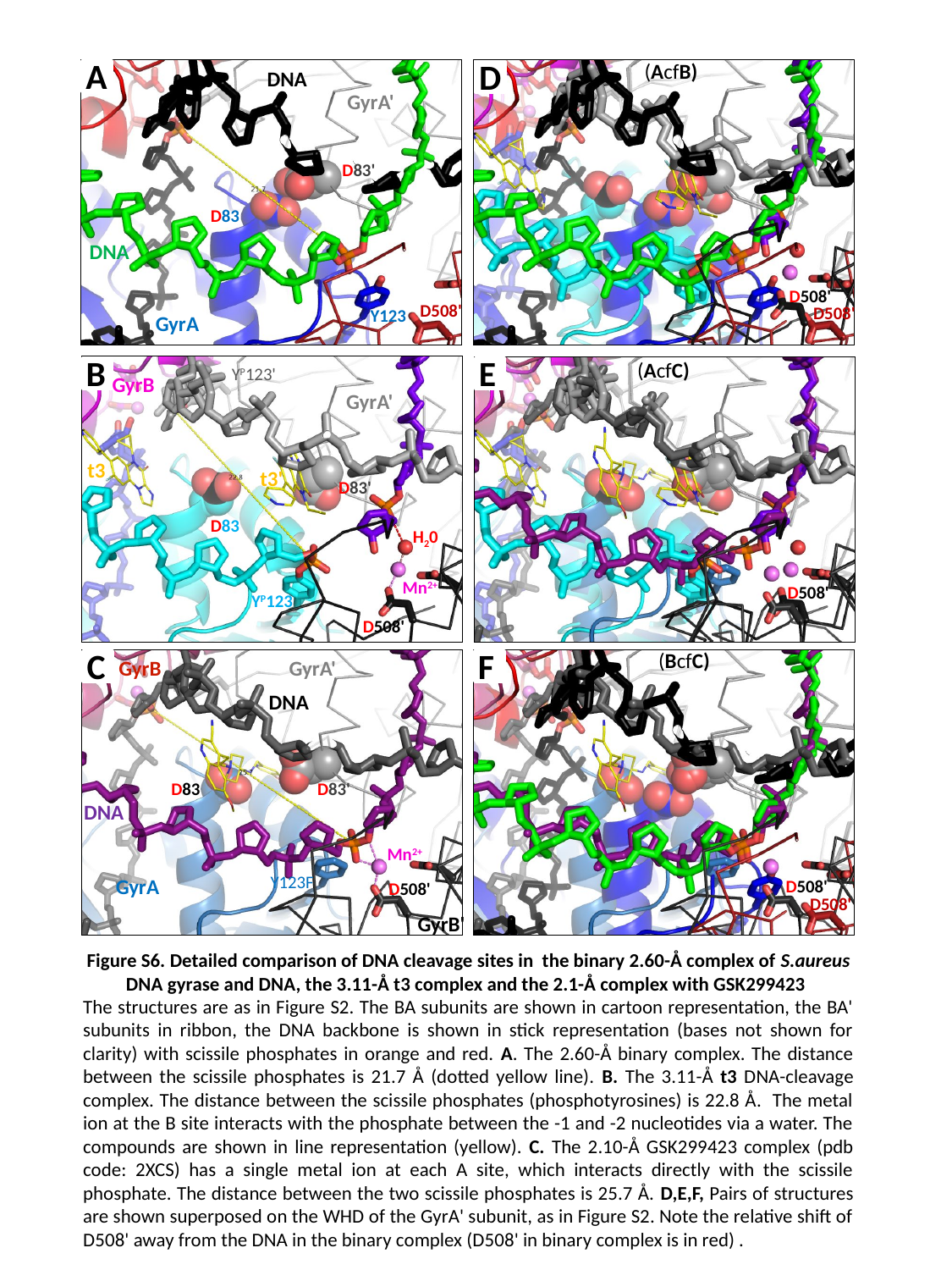

A
D
(AcfB)
DNA
GyrA'
D83'
D83
DNA
D508'
D508'
D508'
Y123
GyrA
B
E
(AcfC)
YP123'
GyrB
GyrA'
t3
t3'
D83'
D83
H20
Mn2+
D508'
YP123
D508'
F
C
(BcfC)
GyrA'
GyrB
DNA
D83
D83'
DNA
Mn2+
Y123F
GyrA
D508'
D508'
D508'
GyrB'
Figure S6. Detailed comparison of DNA cleavage sites in the binary 2.60-Å complex of S.aureus DNA gyrase and DNA, the 3.11-Å t3 complex and the 2.1-Å complex with GSK299423
The structures are as in Figure S2. The BA subunits are shown in cartoon representation, the BA' subunits in ribbon, the DNA backbone is shown in stick representation (bases not shown for clarity) with scissile phosphates in orange and red. A. The 2.60-Å binary complex. The distance between the scissile phosphates is 21.7 Å (dotted yellow line). B. The 3.11-Å t3 DNA-cleavage complex. The distance between the scissile phosphates (phosphotyrosines) is 22.8 Å. The metal ion at the B site interacts with the phosphate between the -1 and -2 nucleotides via a water. The compounds are shown in line representation (yellow). C. The 2.10-Å GSK299423 complex (pdb code: 2XCS) has a single metal ion at each A site, which interacts directly with the scissile phosphate. The distance between the two scissile phosphates is 25.7 Å. D,E,F, Pairs of structures are shown superposed on the WHD of the GyrA' subunit, as in Figure S2. Note the relative shift of D508' away from the DNA in the binary complex (D508' in binary complex is in red) .

## Slide 7
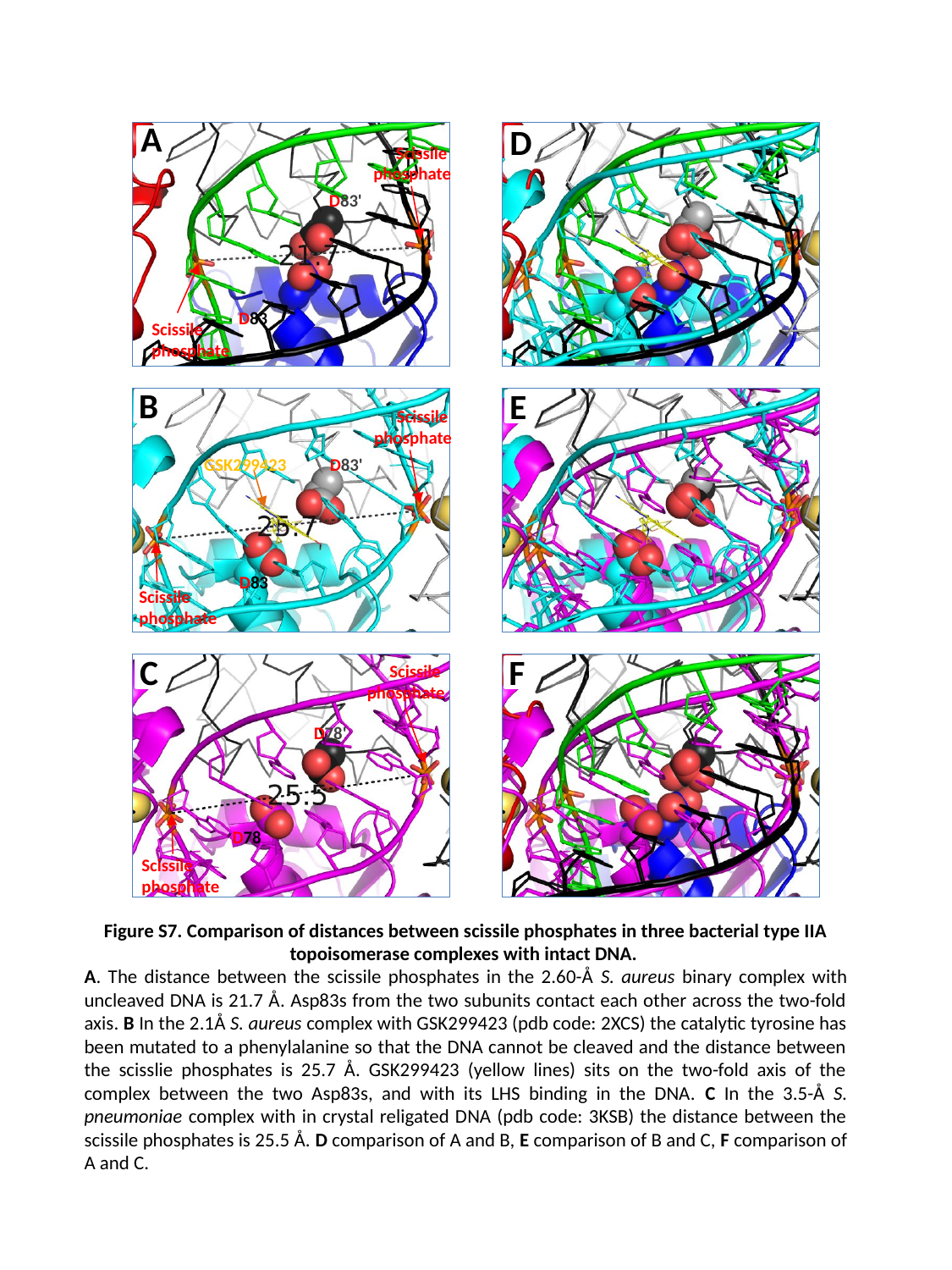

A
D
Scissile
phosphate
D83'
D83
Scissile
phosphate
B
E
Scissile
phosphate
GSK299423
D83'
D83
Scissile
phosphate
F
C
Scissile
phosphate
D78'
D78
Scissile
phosphate
Figure S7. Comparison of distances between scissile phosphates in three bacterial type IIA topoisomerase complexes with intact DNA.
A. The distance between the scissile phosphates in the 2.60-Å S. aureus binary complex with uncleaved DNA is 21.7 Å. Asp83s from the two subunits contact each other across the two-fold axis. B In the 2.1Å S. aureus complex with GSK299423 (pdb code: 2XCS) the catalytic tyrosine has been mutated to a phenylalanine so that the DNA cannot be cleaved and the distance between the scisslie phosphates is 25.7 Å. GSK299423 (yellow lines) sits on the two-fold axis of the complex between the two Asp83s, and with its LHS binding in the DNA. C In the 3.5-Å S. pneumoniae complex with in crystal religated DNA (pdb code: 3KSB) the distance between the scissile phosphates is 25.5 Å. D comparison of A and B, E comparison of B and C, F comparison of A and C.
